# Temporal consistency of behavior trait measurements in guide dogs

**DOI:** 10.3389/fvets.2025.1549360

**Published:** 2025-07-04

**Authors:** Emma K. Hilby, Aaron Rendahl, Jane Russenberger, Madeline Zimmermann, James R. Mickelson, Molly E. McCue

**Affiliations:** ^1^Department of Veterinary Population Medicine, University of Minnesota, St. Paul, MN, United States; ^2^Department of Veterinary and Biomedical Sciences, University of Minnesota, St. Paul, MN, United States; ^3^International Working Dog Registry, San Antonio, TX, United States; ^4^Guiding Eyes for the Blind, Yorktown Heights, NY, United States

**Keywords:** guide dog, behavior consistency, Canine behavior, behavior checklist, behavior prediction

## Abstract

Guide dog organizations have strict criteria to breed, raise, and select dogs to assist people with visual impairments. In collaboration with Dr. James Serpell, several guide dog training organizations developed a scoring tool called the Behavior Checklist (BCL) to evaluate candidate guide dogs. The tool’s use has expanded to the entire assistance dog industry and is rapidly emerging as the standard behavior assessment. Since 2003, Guiding Eyes for the Blind (GEB) has used the BCL to measure individual dogs’ behaviors up to 8 times between puppyhood and final placement. Here, we evaluate the consistency of the BCL over multiple evaluations in a population of 3,969 Labrador Retrievers raised by Guiding Eyes. We grouped BCL evaluations by two methods, factor analysis, and trainer-defined groups, and summarized groupings of behavior in two ways, using mean and lowest scores. We then determined the agreement between pairs of evaluations using kappa statistics and the predictive capacity of early BCL scores to predict later scores using positive and negative predictive values. Evaluations that are similar in nature and those that are scored within 3 to 6 months of one another agree the most. When a dog scores well early in life, they are likely to consistently score well and the dog’s behavior is unlikely to regress over time. We also found that dogs who score poorly early in life either improve their scores on later evaluations with training intervention or are removed from training. One limitation of this data is that dogs who score poorly at early time points are often removed from training and the data from later BCL evaluations is biased toward higher-scoring dogs. Regardless, these data show that the BCL is an effective way to evaluate assistance dog behavior and has some predictive capacity.

## 1 Introduction

Efforts to quantitatively analyze canine behavior have been attempted throughout history. However, unlike physiological traits, which can be measured objectively, behavior is inherently subjective and thus challenging to quantify. Currently, several different behavioral scoring systems are used to describe the behavior of dogs. The Canine Behavioral Assessment and Research Questionnaire (C-BARQ) has been developed for all dogs to assess various behavior attributes, such as aggression, fear, and anxiety (1). The C-BARQ has been used previously to predict guide dog success, however, Guiding Eyes for the Blind (GEB) did not find it adequate for predicting success in their program because the questions the C-BARQ identified as predictive of success were not the primary reasons dogs were being released from their program, therefore there was little utility in using it as their primary behavior evaluation (2). While the C-BARQ has been used by assistance dog organizations, it lacks some important assistance dog-specific behaviors that many organizations want to quantify, such as harness and body sensitivity, stress response, and willingness to work. Behavior is genetically and environmentally complex, and measuring the specific attributes of assistance dog success has been challenging and debated (3). Many researchers have developed questionnaires, utilized aspects of the C-BARQ, or surveyed volunteers raising assistance dogs in training to attempt to capture behaviors of interest. With the development of assistance dog breeding programs, a standardized, consistent, reliable scoring system was needed.

The Behavior Checklist (BCL) is a behavior scoring system initially developed by James Serpell at the University of Pennsylvania, in collaboration with The Seeing Eye, as a validation tool for the C-BARQ (4). GEB led the further development of the BCL with the support and guidance of Dr. Serpell and the involvement of multiple guide and assistance dog organizations (5). The BCL has since been widely adopted and is rapidly emerging as an industry standard. The International Working Dog Registry is a database that stores and analyzes BCL data for assistance dog organizations around the world. The registry currently contains 82,000 BCLs from 109 unique assistance dog organizations, with over 59,000 BCLs submitted from the United States alone (6, 7). The BCL’s definitions are based on outward signs of stress, and the face validity of the BCL was verified using wearables that measured psychological measures of movement and heart rate with a 90% accuracy in predicting BCL scores (8). It also has been instrumental in providing data for calculating estimated breeding values for genetic improvement by identifying replacement breeding dogs most likely to produce progeny possessing the desired behavior traits in guide dogs (6). The 52 BCL items rate aspects of behavior spanning anxiety, fear, aggression, stress, and other behaviors after exposure to a variety of stimuli. These traits can be grouped into categories based on common reasons dogs are released from training programs (“trainer-defined groups”), including emotional composure, resilience, environmental soundness, adaptability, touch sensitivity, willingness, initiative, as well as some miscellaneous items based on definitions for each BCL item, most being on a 1 to 5 scale. GEB utilizes the BCL at several time points, including shortly after weaning, throughout raising, at the start of training, part-way through training, and before client placement. Dogs released from training are typically evaluated at the time of dismissal. Longitudinal BCLs are scored based on observations from two formal tests and multiple observations of similar assessments during the 2–14 months of puppy raising and another set of multiple observations during professional guide dog training. Typically, dogs with consistent moderate or severe reaction to stimuli are released from training programs shortly after evaluation.

The BCL is already widely used as an assistance dog evaluation tool, however, the predictive power and consistency of scores over time have received minimal attention. Previously, a subset of dogs from the Seeing Eye was used to determine if the BCL items accurately measured the underlying emotional state (i.e., BCL construct validity) at its initial development (4). Later a subset of data from GEB was used to analyze the prediction accuracy as the dog ages (9). Investigations into how neonatal and juvenile differences in maternal rearing style and environmental exposures influence important behavioral traits, such as problem-solving, aggression, and fearfulness, have shown that early experiences impact dog career outcomes, despite training intervention (10, 11).

This study examines changes in BCL scores over time in a large study population of Labrador Retrievers bred for assistance work to answer two important questions: (1) how consistent are BCL behavioral measurements over time as the dog matures and advances in their training; and (2) how well do BCL scores on a given evaluation predict the dog’s scores on future BCL evaluations? Knowing which behaviors are heavily modified by the environment will enable age-appropriate training regimens that better shape the desired behaviors and increase assistance dog success rates. Conversely, knowing the behaviors that persist regardless of maturity or training will improve the criteria for the selection of assistance dog candidates. This would allow organizations to make release decisions earlier and avoid spending time and money on dogs that will ultimately be unsuccessful.

## 2 Materials and methods

All analyses were performed using R v4.3.2.

### 2.1 Cohort

15,374 BCL evaluations from 3,969 Labrador Retrievers from GEB were used in this study. Dog birth years span between 2008 and 2020, with most dogs born between 2012 and 2018. All dogs were raised and trained according to GEB protocols. The dogs in this dataset came from 774 litters raised at GEB (168 sires, 298 dams). Not all dogs were evaluated at every time point. For exploratory and confirmatory factor analysis and behavior consistency analysis, 14,485 BCL evaluations from 3,440 Labrador Retrievers were used. A validation cohort was used to validate factor analysis, which consisted of 889 BCLs from 529 Labrador Retrievers from GEB.

### 2.2 Evaluations

At GEB, multiple BCLs, up to 8 assessments, are conducted for each dog starting at 2 months of age. Three types of evaluations are conducted: formal tests, walks in town, and composite impressions from formal training (Table 1). There are 52 items scored on the BCL. Forty-nine BCL items are scored on an ordinal scale of one to five, with one being least favorable and five being most favorable. For each item, the ordinal scores (1–5) are defined with descriptive terms (12). Two BCL items are scored on an ordinal scale of 1–9 and one item is scored on an ordinal scale of 1–6. It is important to note that not all items are scored at each BCL evaluation time point, for example, “dog problems” and “resource guarding” are only evaluated 1–2 times, and thus were not able to be compared at all time points. BCL scorers were all trained at GEB and evaluated for consistency and accuracy of scoring prior to collecting BCL data. Dogs can be released from training at any stage, from puppy test through final training, if they consistently exhibit moderately or severely unfavorable scores for emotional composure, environmental soundness, or house manners despite intervention. Mature dogs with undesirable behaviors undergo detailed review to determine if the next step is release or further remedial training. Generally, the proportion of dogs released for behavior, conformation, or medical reasons is “Puppy Test” (P, approximately 20% of dogs), Puppy Raising (approximately 3% of dogs), and “In for Final Training Test” (IFT, approximately 7% of dogs).

**Table 1:** Descriptions of each BCL evaluation, including average age at evaluation and location.

Evaluation	Age at evaluation	Type of assessment	Location	Description
Puppy Test (P)	7–8 weeks	Formal Test	GEB Canine Development Center	Dogs are brought through a series of novel stimuli (GDBART Puppy Test) in a controlled environment for 12–15 min. Scoring is based on performance from the test and observations recorded from multiple early socialization events prior to the puppy test. At GEB, this test is performed on all dogs in the program and dogs that are the best fit for the program are kept. It informs GEB on the placement in their program or if the dog is better suited for assistance work or another career, such as assistance work at another organization.
Walk and Talk (W)	W1: 4 months W2: 8 months W2b: 10 months W3: 13 months	Walk in Town	Public Indoor or Outdoor age appropriate setting	Dogs are brought through a series of stimuli in an age appropriate public space with their volunteer puppy raiser as their handler and a trainer observing. Most dogs receive two walk and talks, however, dogs with behavioral concerns may be evaluated more frequently. Historically GEB conducted three (W1, W2a, W3) assessments on dogs until 2015, when they switched to two assessments (W1, W2b) due to staffing changes. After 2020, W3 is still occasionally conducted if dogs are called in for final training later than anticipated.
In for Final Training (IFT)	18 months	Formal Test	GEB Training Center	Dogs are brought through a series of novel stimuli (GDBART Test) in a controlled environment for 10–15 min at the GEB training center. Typically, dogs enter the facility on Sunday for final training, spend the weekend in the kennel, and are tested on Tuesday. Besides the PT, this is the only test where the handler is unfamiliar to the dog.
Preliminary Blindfold (PB)	Varies—midway through training	Composite Impressions from Training	Public	Scored by the dog’s instructor mid-way through final training (on average, 3 months after entering final formal training). Based on a formal assessment of the dog performing guide dog tasks with the handler blindfolded plus composite observations from the past month in training.
Final Blindfold (FB) or Released from training	Varies—end of training	Composite Impressions from Training	Public	Scored by the dog’s instructor either when released from training or after formal guide dog training (on average, 3 months after the PB) right before the dog is placed with a client. This evaluation is based on observations over the past month and is conducted similarly to the PB, but with a few added difficulties. This evaluation determines if the dog is ready to start client training.

Each dog had BCL scores collected two to eight times throughout raising and training. The number of evaluations is based on the dog or volunteer raiser’s needs. Not all evaluations are conducted the same way, with modifications made to meet GEB’s needs at each stage of development. Supplementary Table 1 describes the average age at each evaluation time point and the number of dogs who had each evaluation. Each BCL for each dog was labeled as a particular evaluation time point (e.g., a column that stated the evaluation was a “puppy test”). Further filtering was performed to ensure the correct age at evaluation was kept (i.e., a dog who was labeled “puppy test” with an age at BCL of 6 months was excluded from the puppy test sub-cohort). Dogs falling outside of the standard age categories were eliminated from the dataset.

### 2.3 Analyses overview

**Figure 1:**
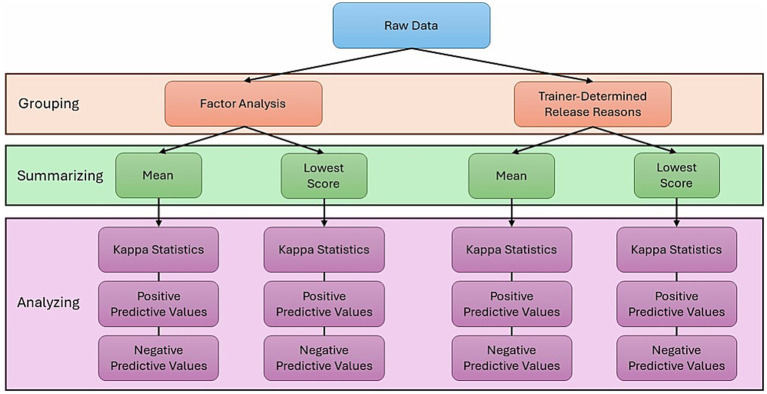
Analysis overview. Raw data was grouped in two ways: factors calculated using factor analysis, and trainer-defined groups, determined by GEB trainers. These groups were then summarized using two methods: arithmetic mean and lowest score within the group of BCL items. Each group-summary pair was analyzed using three different analysis methods: kappa statistics, positive predictive values, and negative predictive values.

Raw BCL data was grouped in two different ways (trainer-defined groups and factor analysis), and data within these groups were summarized in two different ways (mean score and lowest score), creating four separate datasets. Three different analyses were done on each dataset, Kappa statistics, positive and negative predictive values, for a total of 12 sets of results (Figure 1).

#### 2.3.1 Trainer-defined behavior groups

Ten groups of BCL items were defined by guide dog instructors. The groups represent sets of BCL items that capture the characteristic behaviors that commonly result in a dog being released from training at GEB. These groups include Adaptability, Chasing, Emotional Composure, Distraction, Dog Problems, Environmental Soundness, Fear of Heights, Resource Guarding, Touch Sensitivity, and Manners (see Supplementary Table 2). They will be referred to as “Trainer-Defined Groups.” These analyses do not use the individual dog’s outcomes, instead, the analyses use the dog’s BCL scores in each Trainer-Defined Group.

#### 2.3.2 Factor analysis (FA)

Factor analysis is sensitive to missing data. To identify the most robust factors, missing data was imputed on the entire dataset using the “mice” package in R. Imputed data was used only for factor analysis.

To determine a set of factors that were useful across BCL time points, we first did an exploratory factor analysis (EFA) independently on each observation time point (e.g., puppy test, walk and talk, etc.). EFA was performed using the “psych” package in R. Factor values below 0.45 were excluded from EFA results. Most factor loading cutoffs range between 0.3 and 0.5, but the most universally accepted cutoff is 0.40 for satisfactory variables to load onto the primary factor (13). Then, each EFA was tested on all other observation time points to determine the optimal factor groups for the BCL for confirmatory factor analysis (CFA). Finally, CFA was performed using the “lavaan” package in R, and the optimal factors for all evaluation time points were determined using the Comparative Fit Index (CFI, closest value to 1), Tucker-Lewis Index (TLI, closest value to 1), and Root Mean Square Error of Approximation (RMSEA, values between 0.05 and 0.10) (14). The “lavaan” package specifically identifies RMSEA values between 0.05 and 0.08 as a reasonable approximate fit, with anything above 0.10 being a poor fit and anything below 0.05 being a close fit (15). While RMSEA has no universally acceptable values, it should be used in tandem with other values of fit, such as CFI and TLI, to determine which model is the best fit (16). These factors were validated in an independent group of dogs from GEB at the preliminary blindfold (PB) and the final blindfold (FB) evaluation time points. Factors were then named by an expert evaluator based on the items included.

#### 2.3.3 Lowest score vs. mean

Two different scores were used for each subsequent analysis. BCL scores were summarized for each trainer-defined group/factor at each evaluation time point for each individual. The first was the “mean score,” which was calculated from the raw, unimputed score for each BCL item within each trainer-defined group/factor and evaluation time point. The mean score was calculated for each trainer-defined group/factor and time point using the arithmetic mean. The second score is the “lowest score,” which was determined by the lowest raw, unimputed BCL score of all BCL items within each trainer-defined group/factor. The lowest score was calculated for each trainer-defined group/factor and time point. This approach was used to prevent the masking of problem behaviors by other higher scores in the trainer-defined group group/factor.

For all subsequent analyses, custom functions in R were created to calculate kappa statistics, positive predictive values, and negative predictive values. Values were filtered to keep at least 50 dogs per comparison, at least 4 out of the 5 scores required at least 10 dogs, and at least 2 evaluations per dog.

### 2.4 Kappa statistics

Raw, unimputed values were used to calculate kappa analysis. Agreement between scores at different time points was calculated using a Kappa statistic to determine the consistency of scoring over time. This statistic looks at the “raw” scores from each evaluation category and compares them to one another directly, unlike PPVs and NPVs, which categorize scores into two groups based on their value. Kappa were calculated using the following formula:


$$\begin{array}{l} \text{Kappa}=(\text{actual agreement}-\text{expected agreement})/\\ \left(1-\text{expected agreement}\right) \end{array}$$

### 2.5 Predictive values

**Figure 2:**
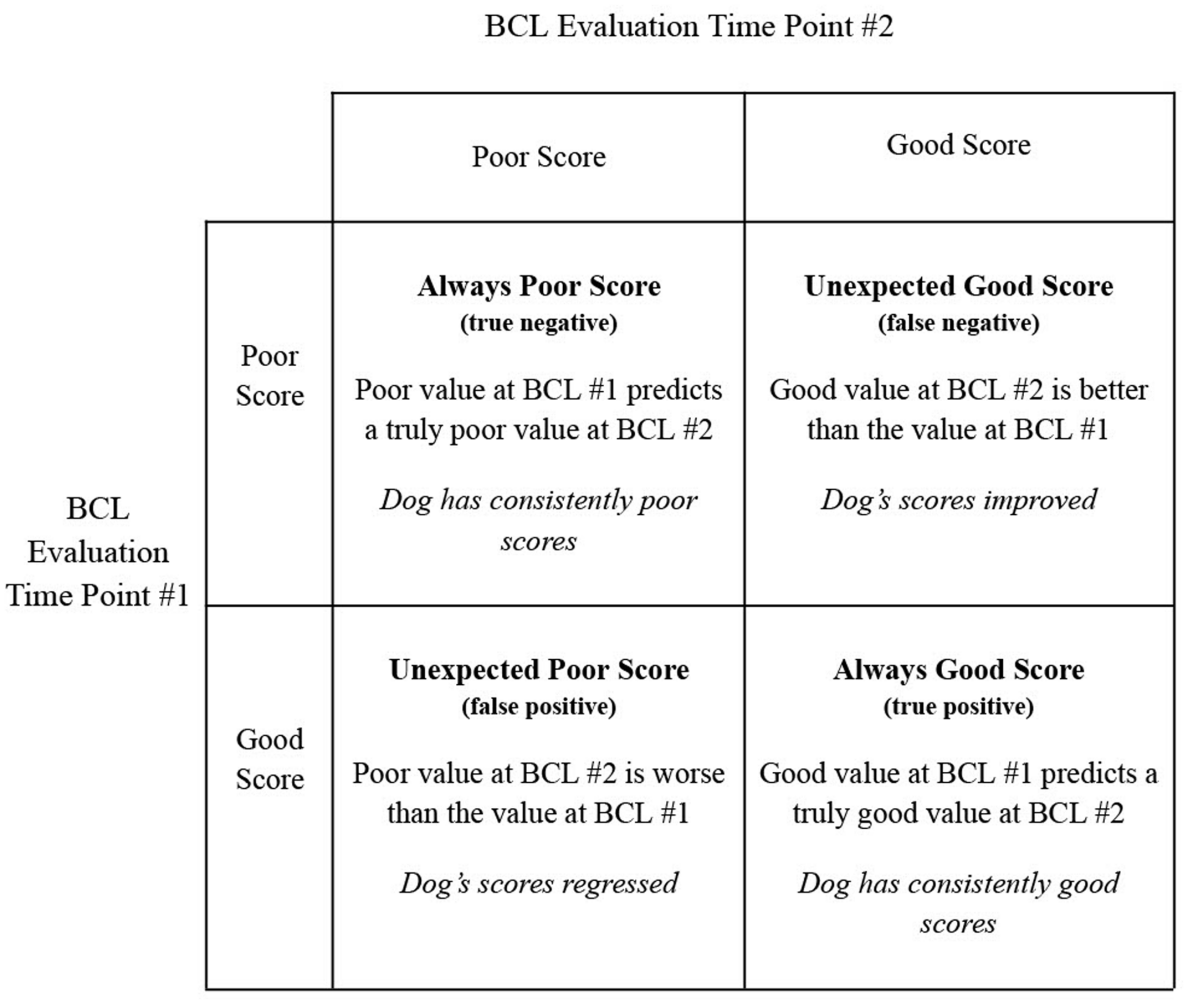
A confusion matrix illustrating the calculations for predictive values. Ideally, a dog is either always good or the score improves–true positives and false negatives, respectively. Each predictive value compares an earlier evaluation to a later evaluation.

Raw, unimputed values were used to calculate all predictive values. Positive and negative predictive values were used to determine how predictive earlier evaluation scores are for later evaluation scores. A confusion matrix best illustrates this idea (Figure 2). Positive and negative predictive values are reported as a percentage; the higher the percentage, the higher the reliability of the positive or negative predictions. Lower percentage values indicate lower reliability of predictive values (i.e., true positives or true negatives are not common).

#### 2.5.1 Positive predictive values

Positive predictive values (PPVs) were calculated to determine how well a favorable score early could predict a favorable score at a later time point. Using the BCL’s 1–5 scale, 1.00–2.99 was defined as a poor test outcome for an item, and 3.00–5.00 was defined as a favorable test outcome. While organizations do consider the dog’s “raw” score, grouping scores into “favorable” and “unfavorable” categories may be a better approach when attempting to predict success or failure. PPVs were calculated using the following formula:


$$\begin{array}{l} \mathrm{PPV}=\text{always good score}/\\ \left(\text{always good score}+\text{unexpected poor score}\right) \end{array}$$

#### 2.5.2 Negative predictive values

Negative predictive values (NPVs) were calculated to determine how well a poor score at an early point could predict a poor score at a later time point. Using the BCL’s 1–5 scale, 1.00–2.99 is a poor test outcome for an item, and 3.00–5.00 is a favorable test outcome. NPVs were calculated using the following formula:


$$\begin{array}{l} \mathrm{NPV}=\text{always poor score}/\\ \left(\text{always poor score}+\text{unexpected good score}\right) \end{array}$$

## 3 Results

### 3.1 Study population

**Figure 3:**
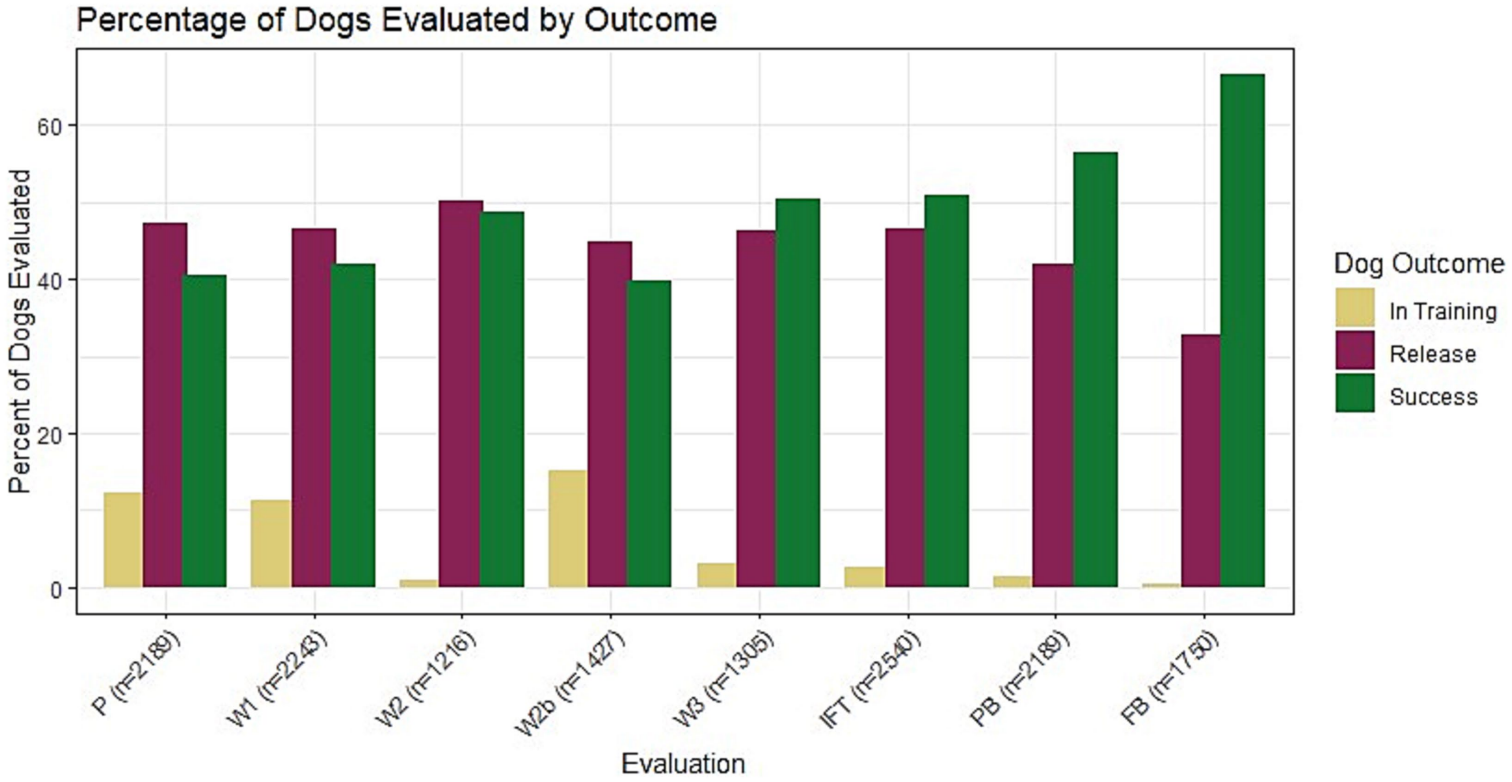
The percentage of dogs that were in training (in training, yellow), released from training (release, purple), or successful (success, green) at each evaluation from Puppy Test (P) to Final Blindfold (FB). The ratio of successful dogs to unsuccessful dogs increases over time from the P to FB, because the worst-performing dogs are released from training periodically throughout the training process. The discrepancy between successful and unsuccessful dogs is the highest at the FB, which biases the data later evaluations toward successful guide dogs.

The number of BCL evaluations each dog had ranged between 2 and 8 assessments, with the mean number of BCL evaluations per dog being 4. The most dogs (n = 2,243) were evaluated at the IFT timepoint, and the least dogs (n = 1,216) were evaluated at the 8-month-old walk and talk (W2) timepoint. Supplementary Table 3 shows items evaluated at each time point and the proportion of dogs evaluated for each BCL item. Not all BCL items were assessed at every time point, with the proportion of dogs evaluated for each BCL item at each time point ranging from 0.00 to 100.00%. The proportion of successful dogs compared to unsuccessful dogs increases as dogs increase in age, indicating that the “worst performing” dogs are being eliminated periodically throughout raising (see Figure 3).

### 3.2 Factor analysis

Exploratory factor analysis (EFA) indicated that the puppy test (P) factors best fit all other evaluations (results not shown). Supplementary Table 4 presents the results of the P EFA. P does not score kinesthetic items, therefore a fifth factor including kinesthetic items based on the IFT BCL was added. Items that were repeated across multiple factors were also removed from successive factors. These five factors were used for subsequent confirmatory factor analysis (CFA) to demonstrate that P factors were adequate at describing other BCL time points. Factors were named by expert evaluators (EH, JR): Resilience, Relationship, Arousal-Activated, Distraction, and Kinesthetic. Supplementary Table 5 describes the results of the CFA with these named factors, including Kinesthetic, for all other evaluation time points. CFA results were validated in an independent group of dogs from GEB (see Supplementary Table 6).

### 3.3 Kappa statistics

For kappa statistics (kappa) and all subsequent analyses, two summative values were used for each factor or trainer-defined group: “mean,” i.e., the arithmetic average of the scores for BCL items within a factor or trainer-defined group, and “lowest score,” i.e., the lowest score of the scores for BCL items within the factor or trainer-defined group. “Mean” was first used as the summative measure for factors/trainer-defined groups because it jointly considers all BCL items within the factor/trainer-defined category. “Lowest score” was also included because it may be valuable for catching moderate to severe reactions to stimuli that may be masked by averaging with higher scoring items within a behavior category. Table 2 summarizes kappa values utilizing the mean and lowest score for the factor groups, and Table 3 summarizes kappa values utilizing the mean and lowest score for trainer-defined groups. Supplementary Tables 7 and 8 display the complete kappa values for factor groupings and trainer-defined groupings, respectively.

**Table 2:** Kappa values calculated for factors using both mean and lowest score.

Type	Group	Mean	Median	SD	Min	Max	Range
Mean	Resilience	0.17	0.07	0.27	−0.08	0.84	0.92
Mean	Relationship	0.17	0.11	0.19	−0.05	0.65	0.69
Mean	Arousal activated	0.24	0.13	0.29	−0.06	0.79	0.85
Mean	Distraction	0.10	0.06	0.15	−0.08	0.59	0.67
Mean	Kinesthetic	0.12	0.07	0.17	−0.04	0.67	0.71
Lowest score	Resilience	0.07	0.04	0.09	−0.01	0.37	0.38
Lowest score	Relationship	0.10	0.06	0.12	−0.02	0.43	0.45
Lowest score	Arousal activated	0.13	0.09	0.12	0.01	0.45	0.44
Lowest score	Distraction	0.08	0.03	0.11	−0.06	0.43	0.49
Lowest score	Kinesthetic	0.10	0.07	0.12	−0.01	0.58	0.60

**Table 3:** Kappa values calculated for trainer-defined groups using both mean and lowest score.

Type	Group	Mean	Median	SD	Min	Max	Range
Mean	Adaptability	0.09	0.07	0.17	−0.11	0.69	0.80
Mean	Chasing	0.10	0.05	0.13	−0.03	0.46	0.49
Mean	Emotional composure	0.17	0.13	0.22	−0.07	0.70	0.77
Mean	Distraction	0.11	0.06	0.15	−0.03	0.50	0.53
Mean	Dog problems	0.05	0.00	0.12	−0.01	0.50	0.51
Mean	Environmental soundness	0.08	0.00	0.19	−0.07	0.66	0.73
Mean	Fear of heights	0.14	0.08	0.14	−0.14	0.42	0.57
Mean	Resource guarding	0.03	0.00	0.09	−0.01	0.40	0.40
Mean	Touch sensitivity	0.11	0.08	0.15	−0.09	0.72	0.82
Mean	Manners	0.14	0.08	0.17	−0.03	0.54	0.57
Lowest score	Adaptability	0.06	0.04	0.14	−0.13	0.52	0.65
Lowest score	Chasing	0.08	0.05	0.09	−0.01	0.38	0.40
Lowest score	Emotional composure	0.09	0.05	0.11	−0.01	0.40	0.41
Lowest score	Distraction	0.10	0.06	0.11	−0.03	0.44	0.47
Lowest score	Dog problems	0.07	0.03	0.10	−0.04	0.38	0.42
Lowest score	Environmental soundness	0.07	0.03	0.10	−0.05	0.39	0.44
Lowest score	Fear of heights	0.14	0.08	0.14	−0.14	0.42	0.57
Lowest score	Resource guarding	0.04	0.00	0.11	−0.01	0.53	0.54
Lowest score	Touch sensitivity	0.10	0.07	0.12	0.00	0.59	0.59
Lowest score	Manners	0.11	0.05	0.13	0.01	0.46	0.45

#### 3.3.1 Factor group lowest score

**Figure 4:**
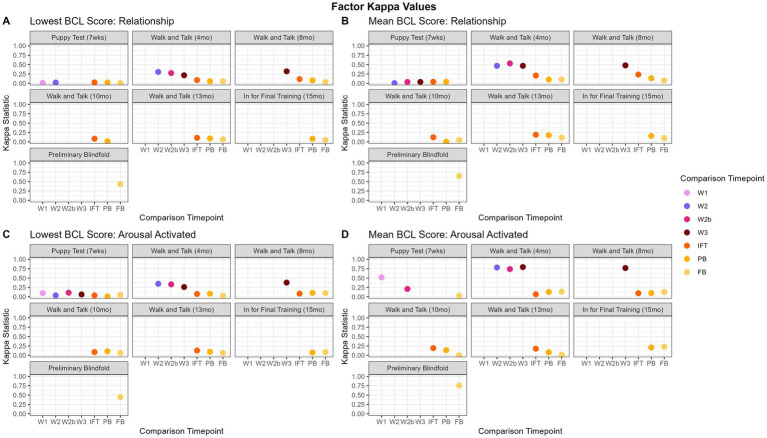
Factor kappa values over time. Panels **(A,B)** show the “relationship” factor and show that mean scores show slightly better agreement between time points. There is a similar trend for “distraction” and “kinesthetic” (data not shown). For “arousal activated,” panels **(C,D)** “resilience” (data not shown), the agreement between W1 and the later walk and talks is much more pronounced when using the mean as the factor summary score. This indicates that mean is a better overall summative value for kappa, and in “arousal activated” and “resilience,” W1 may be all an organization needs to understand how the dog will behave in adolescence for those two behavioral categories.

Kappa scores calculated from the lowest scores ranged from −0.06 to 0.58. For all factors, the highest agreements were between evaluations that were chronologically close together, such as walk and talks to one another and PB to FB (Example “relationship” and “arousal-activated,” see Figures 4A,C). The highest agreement was between PB and FB (“resilience”: 0.37, “relationship”: 0.43, “arousal-activated”: 0.45, “distraction”: 0.43, “kinesthetic”: 0.58).

#### 3.3.2 Factor group mean

Kappa scores calculated from mean scores ranged from −0.08 to 0.84 and are displayed in Figure 4. “Resilience,” “distraction,” and “kinesthetic” had the highest agreement between evaluations that were the same type and chronologically close to each other, such as the walk and talks (0.22 to 0.84) with each other and the PB to the FB (0.59 to 0.67). The highest agreement for “resilience” was between 8-month-old walk and talk (W2) and the 13-month old walk and talk (W3) (0.84), followed by the 4-month-old walk and talk (W1) to W2 and W3 (0.73, 0.72 respectively). The highest agreements for“distraction” and “kinesthetic” were between PB and FB (0.43, and 0.58, respectively). “Relationship” had a similar pattern, with the highest agreements within walk and talks (0.46 to 0.53) and PB to FB (0.65). “Arousal-activated” was similar, with the highest agreement between W1 and W3 (0.79).

#### 3.3.3 Trainer-defined groups lowest score

**Figure 5:**
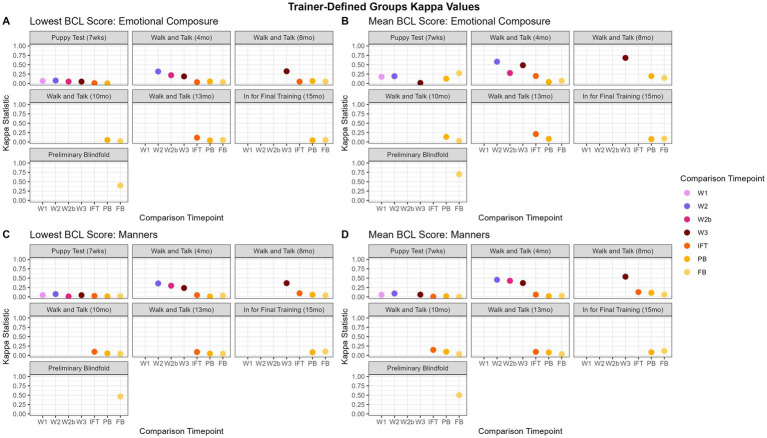
Trainer-defined groups kappa values over time. Panels **(A,B)** show the “emotional composure” trainer-defined group. For the most part, mean has better agreement between evaluations, however, the magnitude of the improvement over the kappa values computed using the lowest score is relatively inconsistent. Panels **(C,D)**, in contrast, show the “manners” trainer-defined group has little difference between the lowest score and mean.

Kappa scores ranged from −0.14 to 0.59 and are displayed in Figure 5. “Adaptability” agreement values were close to zero, except for walk and talks within each other (0.18 to 0.31) and PB agreement with FB (0.52). “Chasing,” “emotional composure,” and “distraction” consistently had the highest agreement between PB and FB (0.38, 0.40, 0.44, respectively). “Touch sensitivity” and “manners” had the highest agreement between chronologically close evaluations, such as PB with FB (0.59, 0.46, respectively), and W2 with W3 (0.22, 0.37, respectively). For “heights,” the highest agreements were between PB and FB (0.42). “Dog problems” and “resource guarding” both had the highest agreement between the PB and the FB (0.38, 0.53 respectively).

#### 3.3.4 Trainer-defined groups mean

Kappa scores ranged from −0.14 to 0.72 and are displayed in Figure 5. For all trainer-defined groups, the highest agreement was between the PB and the FB (0.46 to 0.72). “Adaptability,” “chasing,” “distraction,” “touch sensitivity,” and “manners” had the most agreement with the next evaluation in chronological order (e.g., PB to FB, 0.46 to 0.72). “Emotional composure” was similar in that walk and talks agreed the most with one another (0.27 to 0.68), with the exception being P agreeing with FB (0.27). “Environmental soundness” was fairly inconsistent, with the highest agreement between W1 and W2 (0.66). The highest agreements for “heights” were between the IFT and subsequent evaluations (i.e., PB and FB, 0.33 to 0.38). Agreement between evaluations for “dog problems” were inconsistent, with the highest agreement being between W2 and W3 (0.50). Similarly, agreement between evaluations for “resource guarding” was inconsistent, except for W3 to IFT with an agreement of 0.28 and PB to FB with an agreement of 0.40.

### 3.4 Positive predictive values

As with kappa, “lowest score” and “mean” were used for positive predictive value (PPV) analyses. Overall, PPVs are very high, indicating that, if a dog scores well early in life, it is highly predictive of a good score later in life. Table 4 summarizes PPVs utilizing the mean and lowest score for each factor group, and Table 5 summarizes PPVs utilizing the mean and lowest score for each trainer-defined group. Supplementary Tables 7 and 8 display the complete PPV values for factor groupings and trainer-defined groupings, respectively.

**Table 4:** Positive predictive values were calculated for factor groups using both mean and lowest scores.

Type	Group	Mean	Median	SD	Min	Max	Range
Mean	Resilience	0.98	0.98	0.01	0.96	1.00	0.04
Mean	Relationship	0.97	0.98	0.02	0.91	0.99	0.08
Mean	Arousal activated	0.97	0.98	0.01	0.96	0.99	0.04
Mean	Distraction	0.97	0.97	0.01	0.95	0.98	0.03
Mean	Kinesthetic	0.98	0.98	0.01	0.96	1.00	0.04
Lowest score	Resilience	0.86	0.83	0.07	0.78	0.96	0.18
Lowest score	Relationship	0.94	0.94	0.03	0.88	0.98	0.10
Lowest score	Arousal activated	0.88	0.89	0.03	0.84	0.94	0.10
Lowest score	Distraction	0.94	0.94	0.02	0.90	0.96	0.06
Lowest score	Kinesthetic	0.92	0.92	0.04	0.86	0.97	0.12

**Table 5:** Positive predictive values calculated for trainer-defined groups using both mean and lowest score.

Type	Group	Mean	Median	SD	Min	Max	Range
Lowest score	Adaptability	0.91	0.90	0.05	0.85	0.98	0.13
Lowest score	Chasing	0.96	0.96	0.02	0.92	0.99	0.06
Lowest score	Emotional composure	0.85	0.86	0.09	0.75	0.96	0.21
Lowest score	Distraction	0.90	0.90	0.03	0.86	0.96	0.10
Lowest score	Dog problems	NA	NA	NA	NA	NA	NA
Lowest score	Environmental soundness	0.93	0.92	0.03	0.88	0.96	0.10
Lowest score	Fear of heights	0.98	0.97	0.01	0.97	0.99	0.02
Lowest score	Resource guarding	NA	NA	NA	NA	NA	NA
Lowest score	Touch sensitivity	0.93	0.93	0.04	0.88	0.98	0.10
Lowest score	Manners	0.96	0.96	0.01	0.94	0.98	0.04
Mean	Adaptability	0.99	0.98	0.01	0.97	1.00	0.03
Mean	Chasing	0.97	0.97	0.02	0.94	1.00	0.05
Mean	Emotional composure	0.96	0.96	0.03	0.91	0.99	0.08
Mean	Distraction	0.94	0.93	0.02	0.91	0.98	0.07
Mean	Dog problems	1.00	1.00	NA	1.00	1.00	0
Mean	Environmental soundness	1.00	1.00	0.00	0.99	1.00	0.00
Mean	Fear of Heights	0.98	0.97	0.01	0.97	0.99	0.02
Mean	Resource guarding	NA	NA	NA	NA	NA	NA
Mean	Touch sensitivity	0.98	0.99	0.91	0.96	1.00	0.04
Mean	Manners	0.99	0.99	0.00	0.98	1.00	0.02

#### 3.4.1 Factor group lowest score

**Figure 6:**
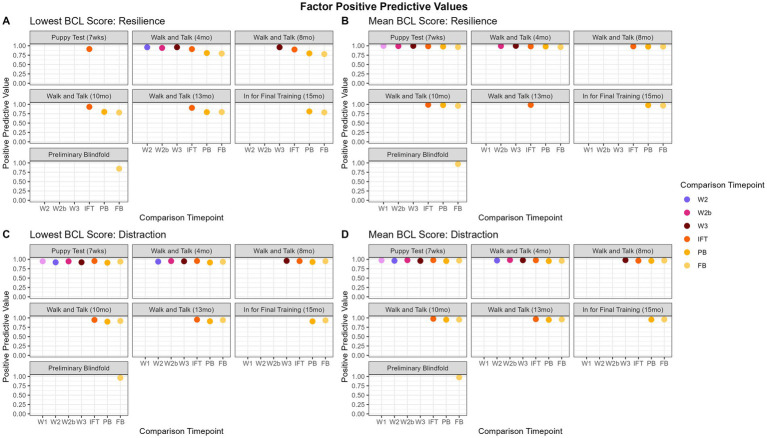
Factor PPVs over time. Panels **(A,B)** show that, while the lowest score is highly predictive of “resilience,” the mean has consistently higher PPVs, especially for later evaluations, indicating that the mean may be a better predictor of PPVs for “resilience.” In contrast, the PPV values do not change significantly for “distraction” **(C,D)** when using mean versus lowest score.

Positive predictive values for the lowest scores ranged from 77.81 to 98.03% and are displayed in Figure 6. Overall, “relationship” and “distraction” had the highest PPV values (summarized in Table 4). For “resilience” and “relationship,” the walk and talk evaluations showed the highest predictive values with one another (92.97 to 98.03%). For “relationship,” walk and talks had the lowest PPV when predicting the IFT BCL. This finding is consistent with the way IFT is conducted in comparison to other evaluations, as the dog is unfamiliar with the handler for the IFT evaluation but is with a familiar handler for all other BCL evaluations. “Arousal-activated” PPVs were consistently lower (84.29 to 93.97%) than PPVs for other factors regardless of which BCL time point was predicted. “Distraction” PPVs were consistently above 90%, with the lowest PPV being 90.00% and the highest being 96.27%. “Kinesthetic” showed a higher PPV value when comparing time points closer to one another, such as the walk and talks (95.38 to 96.31%).

#### 3.4.2 Factor group mean

The range of PPV values for the mean score was 91.48 to 99.88% and are displayed in Figure 6. “Resilience” and “arousal-activated” had consistently high values across the board (95.53 to 99.88%). Still, earlier evaluation time points had slightly lower PPV values when predicting the PB and FB (95.53 to 98.21%). “Relationship” had higher PPV values when predicting later walk and talks from earlier walk and talks (98.94 to 99.19%). “Distraction” had consistently high PPV values (94.96 to 98.37%). “Kinesthetic” had consistently higher PPV values when earlier evaluations predicted evaluations up to W3 (i.e., P to W1; range 98.76 to 99.65%).

#### 3.4.3 Trainer-defined groups lowest score

**Figure 7:**
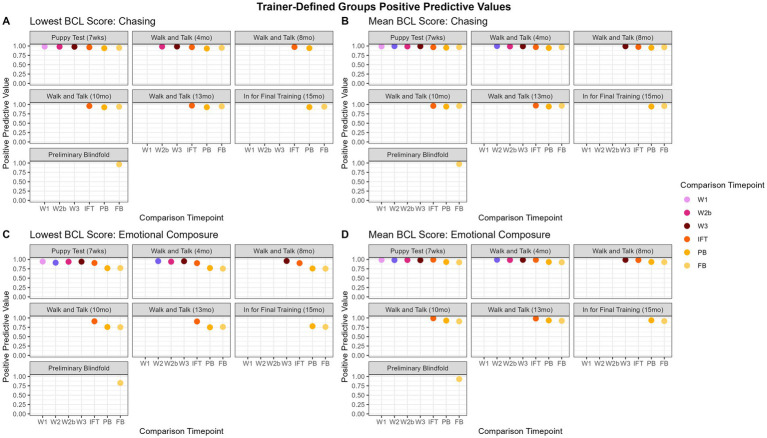
Trainer-defined groups PPVs over time. Panels **(A,B)** show the “chasing” trainer-defined group, indicating that the predictive value does not change between the lowest score and the mean. In contrast, for “emotional composure,” panels **(C,D)**, the mean is a better summative value, as the PPVs decrease is more pronounced for the lowest score over time.

The range of PPV values for the lowest score is 75.00 to 98.79% and are displayed in Figure 7. “Adaptability” had PPVs above 85.31% for P when predicting other evaluations and 91.36% when predicting the PB and the FB. However, there were not enough dogs that met the criteria (see 2.3.3 above) to calculate PPV for W1 through IFT for the adaptability factor. “Chasing” and “emotional composure” were consistently highest when predicting walk and talks within each other (94.00 to 98.58%), but tapered down slightly when predicting FB from PB (96.45 and 82.61%, respectively). “Distraction” was fairly inconsistent and all values were above 86.03%. “Environmental soundness” was also consistently highest when predicting walk and talks within each other (96.80 to 97.53%), but the lowest PPV values were early evaluations to IFT (87.93 to 91.42%). “Touch sensitivity” had the highest predictive values to evaluations close to one another, with the highest PPVs predicting IFT from the walk and talks (94.78 to 95.90%). “Manners” consistently had the highest predictions when comparing early evaluations to PB and FB (96.52 to 97.98%). “Heights,” “dog problems,” and “resource guarding” did not have enough dogs consistently evaluated and enough variability in scores to have PPV scores.

#### 3.4.4 Trainer-defined groups mean

The range of PPV values for the mean was 90.91 to 99.91% and are displayed in Figure 7. “Adaptability” had consistently high scores for all predictions (97.30 to 99.91%). “Chasing” and “emotional composure” had consistently high PPVs, but predicting later evaluations (PB, FB) from earlier evaluations had slightly lower predictions (90.91 to 96.88%) than chronologically closer evaluations (P and walk and talks, 97.01 to 99.78%). “Distraction” was most consistently highest when predicting the IFT from earlier evaluations (97.40 to 98.01%). “Environmental soundness” had consistently high PPVs (99.34 to 99.79%), but no scores through the walk and talks, indicating there were not enough dogs consistently evaluated or with enough variability in scores to produce PPVs. “Touch sensitivity” had consistently high PPVs (97.80 to 99.991%). “Manners” PPVs were consistently high regardless of the time point being compared (97.97 to 99.54%). “Heights,” “dog problems,” and “resource guarding” did not have enough dogs consistently evaluated and enough variability in scores to have PPV scores.

### 3.5 Negative predictive values

As with PPVs and kappa, “lowest score” and “mean” were used for subsequent analyses for negative predictive values (NPVs). Overall, NPVs show that the predictive capacity of the BCL is limited if a dog scores poorly. Looking closely at the differences between evaluation time points, later observations (i.e., the older the dog gets) are more predictive of the scores for subsequent BCLs. Walk and talks are more predictive of each other than other evaluation time points. Table 6 summarizes NPVs utilizing the mean and lowest score for the factor groups, and Table 7 summarizes NPVs utilizing the mean and lowest score for the trainer-defined groups. Supplementary Tables 7 and 8 display the complete NPV values for factor groupings and trainer-defined groupings, respectively.

**Table 6:** Negative predictive values calculated for factor groups using both mean and lowest score.

Type	Group	Mean	Median	SD	Min	Max	Range
Mean	Resilience	0.03	0.00	0.06	0.00	0.21	0.21
Mean	Relationship	0.09	0.03	0.12	0.00	0.35	0.35
Mean	Arousal activated	0.09	0.10	0.08	0.00	0.37	0.37
Mean	Distraction	0.07	0.06	0.08	0.00	0.41	0.41
Mean	Kinesthetic	0.07	0.00	0.12	0.00	0.50	0.50
Lowest score	Resilience	0.25	0.23	0.12	0.08	0.62	0.54
Lowest score	Relationship	0.12	0.10	0.10	0.00	0.43	0.43
Lowest score	Arousal Activated	0.21	0.19	0.10	0.07	0.55	0.48
Lowest score	Distraction	0.12	0.11	0.08	0.00	0.40	0.40
Lowest score	Kinesthetic	0.19	0.18	0.12	0.03	0.55	0.52

**Table 7:** Negative predictive values calculated for trainer-defined groups using both mean and lowest score.

Type	Group	Mean	Median	SD	Min	Max	Range
Mean	Adaptability	0.10	0.00	0.19	0.00	0.53	0.53
Mean	Chasing	0.07	0.04	0.09	0.00	0.33	0.33
Mean	Emotional composure	0.07	0.00	0.11	0.00	0.42	0.42
Mean	Distraction	0.11	0.11	0.11	0.00	0.51	0.51
Mean	Dog problems	1.00	1.00	NA	1.00	1.00	0.00
Mean	Environmental soundness	0.06	0.00	0.14	0.00	0.33	0.33
Mean	Fear of heights	0.14	0.18	0.10	0.00	0.22	0.22
Mean	Resource guarding	NA	NA	NA	NA	NA	NA
Mean	Touch sensitivity	0.09	0.00	0.16	0.00	0.50	0.50
Mean	Manners	0.06	0.00	0.13	0.00	0.44	0.44
Lowest score	Adaptability	0.24	0.10	0.34	0.00	0.73	0.73
Lowest score	Chasing	0.10	0.08	0.09	0.00	0.38	0.38
Lowest score	Emotional composure	0.24	0.23	0.13	0.03	0.62	0.59
Lowest score	Distraction	0.18	0.16	0.10	0.04	0.48	0.44
Lowest score	Dog problems	NA	NA	NA	NA	NA	NA
Lowest score	Environmental soundness	0.11	0.11	0.09	0.00	0.34	0.34
Lowest score	Fear of heights	0.14	0.18	0.10	0.00	0.22	0.22
Lowest score	Resource guarding	NA	NA	NA	NA	NA	NA
Lowest score	Touch sensitivity	0.23	0.19	0.19	0.00	0.56	0.56
Lowest score	Manners	0.13	0.08	0.14	0.00	0.50	0.50

#### 3.5.1 Factor group lowest score

**Figure 8:**
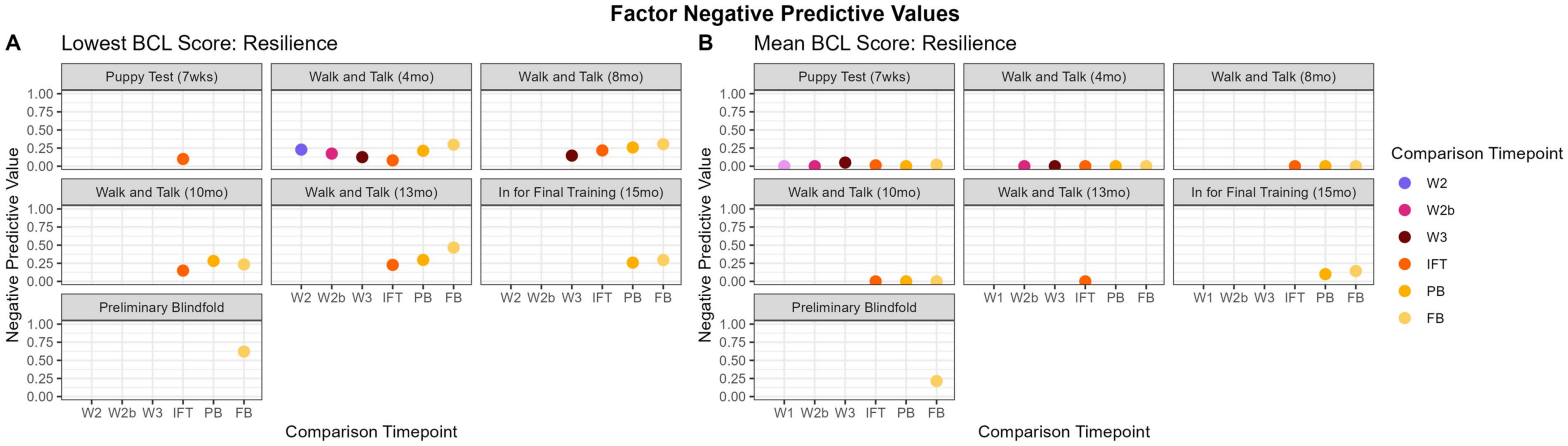
Factor NPVs over time. Panels **(A,B)** display the difference between utilizing the lowest score versus the mean as the summative value for each factor. For “resilience,” most of the values for mean are true zeros, indicating that, if a dog receives a low score (below 2.99), there are other values within that factor that pull the dog’s mean score above 3.

The range of NPV values for the lowest score was 0.00 to 62.11% and are displayed in Figure 8. For “resilience,” the walk and talks had the highest NPV when predicting the PB or FB from earlier evaluations (21.28 to 62.11%). Overall there is an upward trend in NPVs from walk and talks predicting later evaluations to the PB predicting the FB, with the PB having the highest NPV predicting the FB at 62.11%. For “relationship,” NPVs for predicting IFT scores increased from P through W3 with a maximum value of 22.22% between W3 and IFT. The PB had the highest NPV for “relationship” predicting the FB at 43.33%. “Arousal-activated” had fairly consistent NPVs between 7.27 and 37.04% across evaluation comparisons, with the highest NPV predicting the FB from the PB (55.10%). “Distraction” was inconsistent overall, with no scores above 25.53%, except when predicting the FB from the PB (40.41%). “Kinesthetic” had the most consistent NPV values when predicting the IFT and PB from walk and talks, with comparisons staying between 14.81 and 31.43%. The highest “kinesthetic” NPV was predicting the FB from PB, with an NPV of 55.03%. Overall, the PB consistently had the highest NPV for the FB (Table 6).

#### 3.5.2 Factor group mean

The range of NPV values for the mean was 0.00 to 50.00% and are displayed in Figure 8. For “resilience,” the highest NPV was predicting the FB from the PB (21.43%). All other comparisons had near-zero NPVs. Some values were perfect at 0.00% because there were no true negatives (dogs who scored consistently low scores). The highest NPV for “relationship” was 34.62% when predicting the FB from the PB. “Arousal-activated” was fairly consistent over time, staying between 9.09 and 15.38% when comparing P and walk and talks to each other, but was near zero when comparing walk and talks to later evaluations (IFT, PB, FB; 0.00 to 9.09%). The highest NPV value for “arousal-activated” was predicting the FB from the PB (36.73%). “Distraction” was inconsistent overall, with all values below 16.12% except when predicting the FB from the PB (40.85%). “Kinesthetic” had an NPV of 0.00% when predicting walk and talks from earlier evaluations. For “kinesthetic,” IFT was somewhat predictive of PB (33.33%). The highest NPV for “kinesthetic” was predicting the FB from the PB (50.00%).

#### 3.5.3 Trainer-defined groups lowest score

**Figure 9:**
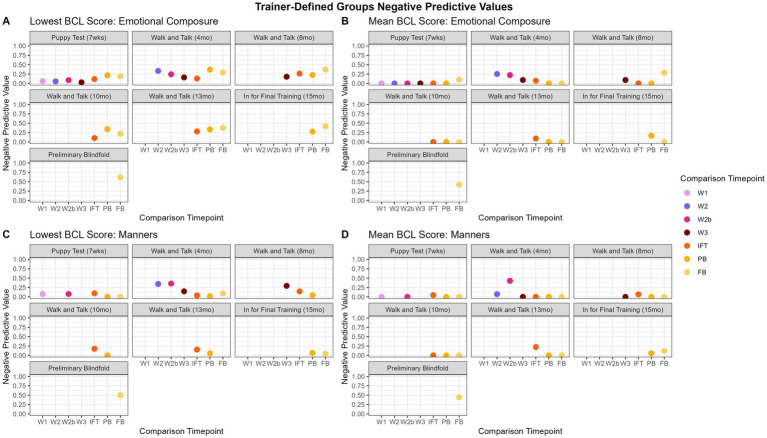
Trainer-defined groups NPVs over time. Figures 8A,B show the “emotional composure” trainer-defined group, which shows that the predictive capacity of NPVs is limited, however, is slightly better when using the lowest score as the summative value over the mean, although there is some predictive capacity for W1 and the other walk and talks for mean. “Manners,” Figures 8C,D, is better predicted using the lowest score, as it appears that the low scores in the “manners” trainer-defined group may be being masked by higher values for the mean.

The range of NPV values for the lowest score was 0.00 to 73.28% and are displayed in Figure 9. “Adaptability” had the highest NPV predicting the FB from PB (73.28%). “Adaptability” did not have enough dogs consistently scored to produce NPVs for the walk and talks and IFT. For “chasing,” the PB was most predictive of the FB (37.5%). The highest NPV for “emotional composure” was for the PB predicting the FB (61.73%). “Distraction” walk and talks had the higher NPVs for predicting the next chronological walk and talk (28.92 to 36.96%), and the highest NPV was the PB predicting the FB (48.29%). The highest predictions for “environmental soundness” were from W3 to IFT (32.00%) and PB to FB (34.26%). “Touch sensitivity” was consistently between 38.10 and 48.39% when predicting PB. The most predictive comparison for “touch sensitivity” was PB predicting FB (56.21%). “Manners” was inconsistent when comparing early evaluations to later, although W1 had some predictive capacity for other walk and talks (34.48 to 35.56%). The highest NPV for “manners” was predicting FB from PB (50.00%). “Heights,” “dog problems,” and “resource guarding” did not have enough dogs consistently evaluated and enough variability in scores to have NPV scores.

#### 3.5.4 Trainer-defined groups mean

The range of trainer-defined group NPV values using mean scores was 0.00 to 52.94% and are displayed in Figure 9. “Adaptability” NPVs were consistently near zero, except when comparing W2b to PB (33.33%), W2b to FB (33.33%), and PB to FB (52.94%). “Chasing” NPVs were consistently highest when comparing walk and talks to PB (12.5 to 33.33%%) and predicting FB from PB (33%). “Emotional composure” and “distraction” were fairly inconsistent, with no clear pattern. PB predicting to FB had the highest NPV of 42.00% for “emotional composure” and 51.22% for “distraction.” “Environmental soundness” had consistently all near-zero NPVs, except when predicting FB from PB (33.33%). “Environmental soundness” NPVs could not be calculated for the walk and talks because there were not enough dogs with scores from items in “environmental soundness” to calculate NPVs. “Touch sensitivity” was highest when predicting IFT from W3 (50.00%) and FB from PB (50.00%). NPVs for “manners” were fairly inconsistent, with most predictions below 11.76%, most of which were 0.00%. “Heights,” “dog problems,” and “resource guarding” did not have enough dogs consistently evaluated and enough variability in scores to have NPV scores.

## 4 Discussion

The BCL scoring tool was created by Serpell and colleagues to analyze the construct validity of the CBARQ questionnaire (2). Subsequently, the BCL was expanded and fine-tuned by GEB in collaboration with Dr. Serpell. The BCL evaluates a variety of important assistance and guide dog behavior traits (5). GEB utilizes the BCL at multiple stages throughout the first 2 years of a dog’s development and training. Current industry practice is to evaluate dogs at least once, if not multiple times, throughout the dog’s training. Up to 52 aspects of behavior can be scored ranging from responses to environmental stimuli, distractions, resilience to stress, touch sensitivity, social manners, desire to work, and walking speed when working. GEB has used the BCL for 20 years, and it has become standard practice in the guide, and now assistance, dog industry to evaluate dogs for training and breeding. Despite this evaluation being used for several years, this paper is the first in-depth analysis of the consistency and predictive capacity of BCL scores at a given time point to predict subsequent BCL scores. Identifying if earlier evaluations can predict later evaluations and which BCL items are consistent over time will aid organizations in removing dogs that are unlikely to be successful despite interventions from training programs sooner. To investigate this completely, we evaluated the relationship between time points using three different statistics (kappa, PPV, NPV), summarizing the data in two different ways: grouping BCL items by trainer-defined groups and data-driven factors and summarizing data within those groupings by mean and lowest score (Figure 1).

Industry practice is to group BCL scores by commonalities within the data to phenotypically evaluate dogs and make more informed career decisions. Groupings are based on expert opinion from GEB trainers who grouped the BCL items into groupings that capture the common reasons for behavioral release. While this is industry practice, it was hypothesized that factor analysis may better categorize BCL behaviors based on their commonality, so a factor analysis was performed based on the data provided. Both the trainer-defined groups and data-driven factors were considered valuable and informative, so both were analyzed. Results indicate that the factors calculated from the data have slightly better predictive capabilities and agreement between BCL evaluations than the trainer-defined groups created by the trainers. This could be because factors are a better summary of BCL score groupings, however, because factors were determined directly from the data, their better performance could be a result of overfitting the data.

Initially, “mean” was used to summarise scores across BCL items within each factor or trainer-defined group because it considers all BCL items within a grouping equally. However, it was clear that a different summary statistic might be valuable to describe the data and best capture poor scores potentially masked by higher scores within the same factor/trained-defined grouping. On further discussion with industry professionals, dogs can be released from training for low scores on a single BCL item or multiple low-scoring BCL items, within the same behavioral category or across multiple behavioral categories. Using the lowest score captures the poorest outcome within a category and ensures that poor scores are not masked by calculating a mean within the behavioral category. Both “mean” and “lowest score” are reported because they describe the data in complementary ways, and add valuable information depending on the outcome (favorable or unfavorable) of interest.

Finally, three different analyses were performed on the datasets created using the two different grouping methods and the two different summative measures described above. The first, and most recognized, is the kappa statistics which determines the agreement between evaluation time points. This is the gold-standard practice when comparing behaviors at different time points. However, how well a given BCL evaluation predicts subsequent evaluations is also an important question, therefore we defined a confusion matrix to calculate predictive values from BCL scores (Figure 2). Predictive values are traditionally used to predict disease susceptibility. However, these predictive measures look at consistently poor and consistently good scores compared to unexpectedly poor and unexpectedly good scores, as illustrated in Figure 2. When a PPV is high, good scores on an earlier BCL predict good scores on later BCLs. When a PPV is low, good scores early on do not guarantee good scores later on. In essence, a dog’s behavior can regress. When a NPV is high, a negative score predicts a negative score on subsequent BCLs, thus suggesting the behavior is unlikely to change and the poor scores will persist on subsequent BCLs. If a NPV is low, this suggests that the behavior is malleable: low scores earlier in life are poor predictors, and it’s likely the score will improve later in life. The caveat with this conclusion is that consistently poor-scoring dogs are removed from training, and therefore from this dataset, so it is impossible to truly know how removed dogs would have scored.

### 4.1 Kappa statistics

Generally, the P does not agree with any other observation time point. However, GEB uses the P to identify the most likely candidate guide dogs and places the remaining puppies in service dog organizations or as pets. At the time this data was collected, 70 to 85% of the puppies born each year remain in the program as candidate guide dogs. The remaining 15 to 30% of puppies released, by default, were filtered from the dataset because they did not have more than one evaluation. It’s important to note that the P is conducted about halfway through the critical period for socialization and further improvement in behavior is expected if properly socialized (17). Walk and talks have more agreement with subsequent walk and talks than other types of evaluations. This makes sense because the walk and talks are carried out in a public setting, unlike the P and IFT. Additionally, walk and talks all occur within a nine-month window over the dogs’ adolescence, typically within 3 to 4 months of one another, and are conducted with their volunteer raiser. For some items, W3 agrees with IFT and the PB with kappa values ranging from 0.00 to 0.30. In general, IFT seems to be a standalone test, similar to the P, “arousal-activated” and “body sensitivity” do agree with the blindfold evaluations, although the agreement is not strong (kappa values ~0.20). The PB and FB agree more than any other pair of evaluations. Two general findings appear when comparing the lowest score to the mean. The first, as seen in Figures 4A,B, is that, for “relationship” (pictured), “distraction,” and “kinesthetic,” both the lowest score and the mean reveal the same general pattern of walk and talks agreeing with one another and PB agreeing with FB, but mean has slightly higher agreement estimates than lowest score. Figures 5C,D demonstrates similar findings in the trainer-defined groups, showing slightly higher agreement using the mean for the “manners” trainer-defined group compared to the lowest score for “manners.” The second, as seen in Figures 4C,D, is that for “resilience” and “arousal-activated” (pictured), is that the walk and talks have a significantly higher agreement when using the mean as the summative score for the factor. This indicates that the dogs do not change much in these two factors throughout the walk and talk evaluations and could suggest that later walk and talks are not needed to capture “resilience” and “arousal-activated,” and that W1 would be sufficient. Figures 5A,B, similarly, has a drastically higher agreement within the walk and talks for the trainer-defined group “emotional composure.” All findings suggest using the mean as the factor summative score is the optimal choice, indicating mean is a better measure of agreement.

### 4.2 Positive predictive values

Positive Predictive Values are generally high overall, regardless of the grouping, summary calculation, or evaluation comparison. This suggests that dogs who score well early on in life continue to score well. The range in PPVs is larger for the lowest scores compared to the range in PPVs for the mean (Figure 6A compared to Figure 6B). This is to be expected because using the lowest score results in a wider distribution of BCL values than the mean score. Overall, using mean scores produces higher PPVs than using the lowest scores. For example, in Figures 6A,B, the lowest score results in lower PPV values when compared to PPV values calculated using the mean score. Thus, PPVs calculated from the mean were more informative, likely due to the mean better capturing the overall signal from the data. Figures 7C,D tells a similar story for trainer-defined groups with “emotional composure,” showing the predictive capacity of earlier BCLs is lower when using the lowest score as the summative value compared to the mean. However, in Figures 6C,D and Figures 7A,B, for “distraction” (factor) and “chasing” (trainer-defined group), there is little difference between using the mean or lowest score for predicting positive outcomes. Overall, the mean is a better predictive measure of good scores on later evaluations, but by a small margin.

### 4.3 Negative predictive values

Negative Predictive Values have a limited capacity for predicting poor outcomes, except when predicting the FB from the PB. This suggests that dogs who score poorly early in life tend to improve their scores to a high enough value to be considered “good” scores (3–5 BCL score), which ultimately is the goal with dogs who are not deemed fit for guide work. This is further complicated by the largest limitation in this dataset: dogs who score the poorest early in life are removed from GEB’s training program. As dogs advance in training, they are progressively released and the data begins to bias towards successful dogs, as seen in Figure 3. This limits the NPV’s ability to predict poor scores when there are fewer and fewer poor scores on later evaluations. The lowest score is a better predictive measure of poor scores than the mean. Additionally, the PB is fairly consistent at predicting a low score for the FB, which may indicate that release decisions can be made if a dog scores poorly on the PB. NPVs are calculated by categorizing scores into “poor” and “good” based on a cutoff of 2.99, with scores below 2.99 being considered a “poor” score and above 3 being a “good” score. Utilizing the mean as the summative value may be masking truly poor scores in these categories, as seen in Figures 8, 9C,D. The NPVs for the mean are almost all near zero, which is again a consequence of the structure of the data. Because dogs with poor scores are often released, the dataset has very few individuals with initial poor scores that remain in the dataset therefore there are a limited number of dogs with poor scores on early evaluations that have poor scores on later evaluations resulting in few data points to constitute a useful NPV. Interestingly, for some of the trainer-defined group groupings, such as “emotional composure” in Figures 9A,B, it appears mean score still results in somewhat useful NPVs, although these NPVs are less informative than NPVs calculated using the lowest score. Utilizing the lowest score as the summative value appears to, overall, resulting in more informative NPVs overall. This does not mask a single poor score in any behavior category but rather uses it as the value for that behavior, which may be beneficial for organizations to use if they are trying to predict poor outcomes in dogs.

### 4.4 Predictive capacity of the BCL

Our results indicate that the BCL is highly predictive of behavior if a dog is exhibiting desired behavior traits; i.e., dogs that have favorable behavioral characteristics tend to continue to be stable in those desired characteristics over time (i.e., dogs do not typically regress from good behaviors). This could be due to positive behaviors being reinforced consistently throughout development and training. Predicting poor behavior is less clear, likely due to the heavy environmental intervention imposed on dogs-in-training, and the limitations in this dataset. Despite this limitation, this dataset does show that a proportion of dogs who score poorly improve over time, but it is unknown if the scale of that improvement is enough for dogs to ultimately succeed in guide dog programs. Overall, the mean appears to be a better predictor of high scores and agreement between scores at different time points, but the lowest score is a better predictor of poor score outcomes.

### 4.5 Behavior consistency

Behavior is one of the two broad categories, along with health, for which dogs are released from training organizations, with some reporting up to 75% of dogs failing due to behavioral problems (18). Behavior consistency and its prediction are areas of interest in both pet and assistance dog populations. Behavioral consistency in several canine populations has been analyzed previously. Fratkin et al. (19) did a comprehensive meta-analysis of personality consistency in dogs and determined that shorter intervals between behavior measurements, consistency of evaluation, and assessment similarities were all factors that contributed to personality consistency (19). Age was one of the most important factors as well, as older dogs tend to be more stable in personality than younger dogs (19). Our results are consistent with this finding, that both similarities of assessment and older assessments tended to be in more agreement and more predictive of one another. Kappa statistics best illustrate this idea, as the highest agreement between evaluations was within the walk and talks and the blindfold evaluations. For factor PPVs, “resilience,” “relationship,” and “kinesthetic” are slightly less consistent than “arousal-activated” and “distraction,” indicating the latter two behaviors may not change as much over time. For trainer-defined group PPVs, “emotional composure” and “adaptability” are slightly less consistent than the remaining trainer-defined groups, which also indicates these behaviors are more malleable over time. For factor and trainer-defined group NPVs, most values are inconsistent over time, indicating that lower-scored items are not consistently low and that training interventions frequently improve undesired behaviors. The exceptions to this are “kinesthetic” (factor), “environmental soundness” (trainer-defined group), and “distraction” (trainer-defined group), which have consistently low scores over time. While the NPVs for these three groupings are relatively low overall, the data still suggests that these behaviors are less likely to improve over time.

Behavior prediction is not a new area of research in the assistance dog industry, where there are many evaluations that researchers and organizations have developed to attempt to quantify and predict important attributes of assistance dog success. A considerable amount of focus has been given to predicting success from early evaluations. Guide Dogs for the Blind, Guide Dogs UK, and Canine Companions have all attempted to utilize different evaluations between 7 and 9 weeks of age to predict success (20, 21). Others have created later evaluations, when temperament stabilizes, to attempt to predict behavioral outcomes, such as Marcato et al. (22) who created a novel assistance dog test battery based on several assistance dog behavior evaluations, which was conducted 3 weeks post-training induction and 10 weeks post-training induction, similar to the PB and FB (22). Prediction of BCL outcomes has been successfully done using sensor systems monitoring heart rate in guide dog puppies at GEB and comparing heart rate variability and motion data to BCL score outcome, but sensor systems are still in development for wide application within the assistance dog industry and may be impractical to implement financially and the technology is not readily available for use (23). While these studies all looked at predicting success as assistance dogs, the understanding of how those behaviors develop and change over multiple evaluations in a dog’s training life was not studied. Most conducted a puppy evaluation or an adolescent evaluation, then a later adulthood evaluation, or just one early evaluation and longitudinally followed the dog’s final outcome. Understanding how behaviors develop, change, and are affected by the environment is critical in understanding when a behavior is worth applying intervention to or whether a dog will ultimately not improve. The true power of the BCL is that it evaluates behaviors that organizations really care about throughout training and has been adopted almost universally by the assistance dog industry, making genetic prediction a possibility for small and large organizations alike.

### 4.6 Implications

Results from the PPVs and NPVs indicate that the BCL has predictive capacity for assessments that are similar to one another, both in the manner they are conducted and in timing, such as walk and talks and Blindfold tests. Additionally, predictive values increase over time becoming more informative as dogs mature, suggesting early behavior is malleable. The high PPVs compared to lower NPVs may be due to dogs scoring poorly early on, effective intervention being applied, and dogs improving in those areas which results in a higher score. This shows that socialization, training, and behavioral intervention are improving dog behavior, which is ultimately the goal for organizations. Less consistent behaviors, with a wider range of PPVs and NPVs, indicate that those behaviors are not as consistent throughout adolescence, and can improve or regress over time. Behaviors such as “kinesthetic,” for example, have consistent scores over time, which can indicate that the behavior may not improve with intervention. This is also supported by the kappa statistic, showing that assessments that agree can also be predictive of each other. Since earlier walk and talk evaluations are good predictors of later walk and talks, some decisions can be made regarding dogs who exhibit undesired behaviors early on during early walk and talk evaluations, and later walk and talks may not be necessary if they are giving the same or similar information. W1 may be all an organization needs to get an idea of if a dog is behaviorally sound (or needs intervention and a management plan) during adolescence if they do not have the resources to perform successive walk and talks. Subsequently, dogs who exhibit undesired behaviors in the PB may be subject to an earlier release, as the most predictive evaluation pairing is the PB to the FB. This is supported by previous work on behavioral consistency in dogs, as evaluations that are similar to each other are more consistent and reliable than those that are different (19). Marcato et al. (22) found that an evaluation later in training is more predictive of assistance dog success than an evaluation earlier in final training, but did not compare the agreement between the two evaluations, just their success prediction capacity (22). Because the P and IFT are similar in structure but far away in time, they are essentially standalone evaluations that are difficult to compare to other behavior evaluations such as the walk and talks or the PB compared to the FB, which are all performed within months of each other and are conducted in the same way for the same dog each time. The nature of these evaluations is also important to consider—for example, the IFT is utilized at a specific age when the dog is mature and is the only evaluation where the dog is completely unfamiliar with their handler. This gives organizations valuable insight into the dog’s adaptability to an unfamiliar person and its ability to cope with stress, which is not necessarily evaluated on any other BCL evaluation.

Behavior is fundamental for assistance dog success, as dogs must be behaviorally sound in stressful environments and be able to perform complex tasks in unpredictable circumstances. The BCL has been used to both predict behavior and make decisions about dog placement, and more recently, has been used for genetic selection. Specifically, BCL data has been translated into estimated breeding values (EBVs) to allow for informed breeding decisions in assistance dog populations. While many of the BCL behavior traits that are heritable, such as BCL item “poor self-modulation” (h2 = 35%) and “relationship skills” (h2 = 43%), they are still subject to environmental influences and are therefore malleable and can be changed with training or other interventions (3, 6). Despite complex gene–environment interactions, GEB utilizes the BCL for several EBVs and has successfully used genetic selection to improve dogs behaviorally over time (6).

### 4.7 Limitations

As mentioned above, the largest limitation of this study and others using real-world data gathered from assistance dog organizations is that most organizations regularly release puppies and dogs in training over time due to poor scores. Thus, the test becomes more biased towards success as dogs age, as only dogs that have the potential to be suitable for assistance work, and thereby high scores, remain in the dataset. Figure 3 shows the percentage of successful dogs, versus those released, at every evaluation, depicting the bias that increases as dogs age. Practically, it is not financially feasible to keep dogs that will ultimately be released in the program to continue to behaviorally test them at each test point, so this limitation is likely to be present in any real-world dataset from the assistance dog industry. Additionally, not all BCL evaluations are conducted in the same way (i.e., P and IFT are in a controlled room environment, all others are in real-world environments), so there are inherent differences in the environments that were not accounted for in this analysis to maximize external validity.

## 5 Conclusion

The BCL is a scoring system used by the assistance dog industry to assess behavior at various stages throughout training. The BCL was initially developed by guide dog organizations to evaluate dog behavior and has quickly become the industry standard. The present study indicates that BCL has an excellent predictive capacity when a dog scores well in early assessments, especially if the mean is used as the summarizing value for behavior groupings. Those BCLs that are similar in structure, setting, and age at evaluation are better predictors of success on subsequent evaluations, while those comparing BCL evaluations that are different in structure, setting, and further apart in age are more challenging to compare directly. Factor analysis groups effectively describe the underlying behavior items on the BCL. The lowest score is the best way to summarize values for groupings if evaluating poor-scoring behavior, however, prediction is limited, likely due to dogs improving over time. It is still unknown if the scale of that improvement is enough for dogs to ultimately succeed in guide dog programs. The BCL is demonstrated to have predictive capacity and some consistency throughout training.

### 5.1 Future work

The BCL is informative over time in this population of guide dogs at GEB. Future work should assess the predictive capability in other types of assistance dogs, such as other guide dog populations or service dog populations. Organizations would also benefit from further understanding of which socialization and training protocols are the most effective interventions in providing more successful dogs possessing the desirable behavior for their intended work.

## Data Availability

The raw data supporting the conclusions of this article will be made available by the authors. Please contact Jane Russenberger (jane.russenberger@iwdr.org) to request the raw data.
