# Supplementary material for: Temporal consistency of behavior trait measurements in guide dogs
Source: Front Vet Sci. 2025 Jul 4;12:1549360. doi: 10.3389/fvets.2025.1549360 (PMC12272753; doi:10.3389/fvets.2025.1549360)
Supplement: Supplementary file 1 [file Data_Sheet_1.pdf]

***Supplemental Table 1.*** Descriptions of each BCL evaluation, including average age at evaluation and location.

| Evaluation | Age at Evaluation | Type of Assessment | Location | Description |
|---|---|---|---|---|
| Puppy Test (P) | 7-8 weeks | Formal Test | GEB Canine Development Center | Dogs are brought through a series of novel stimuli (GDBART Puppy Test) in a controlled environment for 12-15 minutes. Scoring is based on performance from the test and observations recorded from multiple early socialization events prior to the puppy test. At GEB, this test is performed on all dogs in the program and dogs that are the best fit for the program are kept. It informs GEB on the placement in their program or if the dog is better suited for assistance work or another career, such as assistance work at another organization. |
| Walk and Talk (W) | W1: 4 months<br>W2: 8 months<br>W2b: 10 months<br>W3: 13 months | Walk in Town | Public Indoor or Outdoor age appropriate setting | Dogs are brought through a series of stimuli in an age appropriate public space with their volunteer puppy raiser as their handler and a trainer observing. Most dogs receive two walk and talks, however, dogs with behavioral concerns may be evaluated more frequently. Historically GEB conducted three (W1, W2a, W3) assessments on dogs until 2015, when they switched to two assessments (W1, W2b) due to staffing changes. After 2020, W3 is still occasionally conducted if dogs are called in for final training later than anticipated. |

| | | | | |
|---|---|---|---|---|
| In for Final Training (IFT) | 18 months | Formal Test | GEB Training Center | Dogs are brought through a series of novel stimuli (GDBART Test) in a controlled environment for 10-15 minutes at the GEB training center. Typically, dogs enter the facility on Sunday for final training, spend the weekend in the kennel, and are tested on Tuesday. This is the only test, besides the PT, where the handler is unfamiliar to the dog. |
| Preliminary Blindfold (PB) | Varies - midway through training | Composite Impressions from Training | Public | Scored by the dog's instructor mid-way through final training (on average, 3 months after entering final formal training). Based on a formal assessment of the dog performing guide dog tasks with the handler blindfolded plus composite observations from the past month in training. |
| Final Blindfold (FB) or Released from training | Varies - end of training | Composite Impressions from Training | Public | Scored by the dog's instructor either when released from training or after formal guide dog training (on average, 3 months after the PB) right before the dog is placed with a client. This evaluation is based on observations over the past month and is conducted similarly to the PB, but with a few added difficulties. This evaluation determines if the dog is ready to start client training. |

***Supplemental Table 2.*** Number of dogs assessed and average age of evaluation for each evaluation time point.

| Type of Assessment | Observation | n | Min Age | Max Age | Mean Age |
|---|---|---|---|---|---|
| Formal Test | P | 1815 | 7.00 wks | 8.00 wks | 7.75 wks |
| Walk in town | W1 | 2243 | 3.53 mo | 5.10 mo | 4.35 mo |
| Walk in town | W2 | 1216 | 7.10 mo | 9.10 mo | 8.26 mo |
| Walk in town | W2b | 1427 | 9.13 mo | 11.17 mo | 10.20 mo |
| Walk in town | W3 | 1305 | 12.17 mo | 14.23 mo | 13.25 mo |
| Formal Test | IFT | 2540 | 14.27 mo | 18.27 mo | 16.71 mo |
| Composite Impressions from Training Dog | PB | 2640 | 15.53 mo | 30.17 mo | 20.63 mo |
| Composite Impressions from Training Dog | FB | 2188 | 17.73 mo | 32.47 mo | 22.68 mo |

***Supplemental Table 3.*** Release Reason groupings identified by trainers as the most common reasons dogs are released from guide dog training, grouped by similarity.

| Release Reason Bin | BCL Items |
|---|---|
| Adaptability | Separation Anxiety<br>Handler Attachment<br>Kennels Poorly |
| Chasing | Chases Animals<br>Movement Excites |
| Emotional Composure | Fear of New Places and Situations<br>Active Response to Stress<br>Passive Response to Stress<br>Self Modulation |
| Distraction | Dog Distraction<br>Olfactory |
| Dog Problems | Fear of Dogs |

| | |
|---|---|
| | Dog Aggression<br>Dog Aggression On Leash<br>Dog Aggression Off Leash |
| Environmental Soundness | Noise Fear<br>Fear of Novel Objects<br>Underfootings<br>Fear of Traffic<br>Fear of Strangers<br>Fear of Blowing Fan<br>Riding in Vehicle |
| Fear of Heights | Stair Concern |
| Resource Guarding | Possession Aggression<br>Resource Guarding towards Dogs |
| Touch Sensitivity | Yields Space<br>Harness Sensitivity<br>Body Sensitivity |
| Manners | House Manners<br>Social Manners<br>Housebreaking |

***Supplemental Table 4.*** Behaviors evaluated on the Behavior Checklist at each time point, from the puppy test through the final blindfold. These are defined by the percentage of dogs not evaluated for that item (% NA). Percentages are highlighted based on the rate of missing values, 0.00 to 24.99% missingness is the lightest red, and 75.00 to 100.00% missingness is the darkest red.

| BCL Item | PT | W1 | W2 | W2b | W3 | IFT | PB | FB |
|---|---|---|---|---|---|---|---|---|
| # Dogs Scored (Total) | 1815 | 2243 | 1216 | 1427 | 1305 | 2540 | 2640 | 2188 |
| Anxious in Unfamiliar Locations | 0.06 | 0.27 | 0.08 | 0.14 | 0.23 | 0.98 | 9.64 | 9.37 |
| Noise Sensitivity | 0.00 | 0.13 | 0.00 | 0.28 | 0.23 | 0.91 | 1.14 | 2.00 |
| Fear of Novel Objects | 0.17 | 0.40 | 0.16 | 0.63 | 0.31 | 1.89 | 9.82 | 10.00 |
| Fear of Underfootings | 0.06 | 0.22 | 0.33 | 0.28 | 0.23 | 0.94 | 1.32 | 2.29 |
| Fear of Dogs | 1.32 | 0.18 | 0.08 | 0.14 | 0.15 | 1.50 | 1.19 | 2.57 |
| Fear of Stairs | 3.25 | 0.31 | 0.82 | 0.21 | 0.15 | 97.52 | 1.74 | 2.23 |

| | | | | | | | | |
|---|---|---|---|---|---|---|---|---|
| Fear of Traffic | 98.73 | 0.53 | 0.74 | 0.42 | 0.23 | 98.31 | 2.01 | 2.23 |
| Separation Anxiety | 0.33 | 0.04 | 0.08 | 0.07 | 0.23 | 98.39 | 1.82 | 2.57 |
| Hyper-Attachment | 14.33 | 0.31 | 0.16 | 0.07 | 0.15 | 96.10 | 2.65 | 2.11 |
| Fear of Strangers | 0.66 | 0.18 | 0.00 | 0.28 | 0.23 | 1.97 | 1.19 | 2.69 |
| Body Handling | 1.82 | 0.36 | 0.41 | 0.63 | 0.23 | 3.35 | 1.78 | 2.17 |
| Retreats when Reached For | 11.85 | 0.80 | 1.73 | 1.68 | 1.07 | 3.78 | 10.74 | 11.43 |
| Harness Sensitivity | 3.09 | 53.10 | 34.45 | 11.00 | 20.08 | 10.95 | 1.23 | 2.00 |
| Avoidance of Blowing Fan | 12.56 | 10.48 | 17.43 | 11.42 | 15.33 | 13.03 | 24.30 | 24.57 |
| Body Sensitivity | 11.85 | 8.16 | 13.90 | 7.99 | 11.42 | 11.65 | 23.57 | 24.57 |
| Anxious Riding in Vehicles | 98.90 | 6.33 | 12.17 | 6.66 | 9.20 | 97.87 | 15.40 | 17.14 |
| Inhibited when Stressed | 0.39 | 0.31 | 0.82 | 0.07 | 0.46 | 1.06 | 1.69 | 2.29 |
| Activated when Stressed | 0.05 | 0.18 | 0.25 | 0.00 | 0.31 | 0.91 | 1.69 | 2.57 |
| Excitable | 0.00 | 0.04 | 0.41 | 0.21 | 0.08 | 0.83 | 0.96 | 2.11 |
| Slow to Return to Productive Emotional State | 0.00 | 0.13 | 0.41 | 0.00 | 0.08 | 1.22 | 1.28 | 2.57 |
| Fidgety when Handler is Idle | 2.87 | 0.31 | 0.33 | 0.35 | 0.69 | 5.75 | 9.68 | 10.23 |
| Fear of Elevated Areas | 100 | 100 | 100 | 100 | 100 | 100 | 100 | 100 |
| Barks Persistently | 0.17 | 0.22 | 0.66 | 0.28 | 0.61 | 1.85 | 9.78 | 9.94 |
| High Energy | 0.06 | 0.13 | 0.25 | 0.14 | 0.15 | 1.38 | 1.28 | 2.23 |
| Lacks Focus | 0.22 | 0.40 | 0.66 | 0.07 | 0.84 | 1.85 | 0.91 | 2.17 |
| Movement Excites | 0.17 | 0.45 | 0.41 | 0.21 | 0.38 | 1.14 | 9.50 | 9.09 |
| Chasing Animals | 91.18 | 1.83 | 2.96 | 2.03 | 2.07 | 91.46 | 1.10 | 1.66 |
| Dog Distraction | 1.43 | 0.27 | 0.66 | 0.42 | 0.61 | 1.02 | 9.32 | 8.29 |
| Olfactory | 0.00 | 0.09 | 0.49 | 0.28 | 0.31 | 1.02 | 0.87 | 1.43 |
| Scavenges | 2.20 | 0.09 | 0.33 | 0.14 | 0.31 | 43.66 | 1.51 | 2.06 |
| Inappropriate Behavior in the | 93.39 | 5.75 | 10.44 | 5.47 | 8.20 | 23.74 | 58.38 | 52.00 |

| | | | | | | | | |
|---|---|---|---|---|---|---|---|---|
| Home | | | | | | | | |
| Lacks Initiative | 6.06 | 7.22 | 13.57 | 5.75 | 10.42 | 55.83 | 9.91 | 6.34 |
| Not Willing | 2.81 | 0.13 | 0.33 | 0.21 | 0.31 | 6.14 | 9.82 | 10.00 |
| Resource Guarding Towards People | 0.17 | 0.27 | 0.25 | 0.42 | 0.23 | 1.18 | 1.46 | 2.69 |
| Aggression Towards Strangers | 0.39 | 0.18 | 0.16 | 0.28 | 0.00 | 1.73 | 1.23 | 2.74 |
| Aggression Towards Dogs On Leash | 23.25 | 22.07 | 37.17 | 16.47 | 32.80 | 67.40 | 32.98 | 33.26 |
| Aggression Towards Dogs Off Leash | 23.20 | 22.07 | 37.17 | 16.40 | 32.95 | 27.09 | 33.03 | 33.20 |
| Resource Guarding Towards Dogs | 36.86 | 39.14 | 63.98 | 24.95 | 56.70 | 86.42 | 47.05 | 46.46 |
| Inappropriate Elimination | 96.09 | 0.31 | 0.16 | 0.35 | 0.69 | 1.14 | 1.42 | 2.86 |
| Socially Inappropriate Behavior with People | 4.90 | 0.67 | 1.64 | 1.26 | 1.07 | 7.24 | 8.41 | 8.97 |
| Inconsistent | 0.61 | 0.17 | 0.25 | 0.42 | 0.38 | 1.73 | 1.92 | 2.00 |
| Handler-Dog Team | 0.28 | 0.26 | 0.33 | 0.49 | 0.23 | 1.81 | 9.46 | 7.89 |
| Relationship Skills | 100 | 100 | 100 | 100 | 100 | 100 | 100 | 100 |
| Comparison Rating | 0.11 | 0.22 | 0.25 | 0.49 | 0.15 | 1.10 | 9.73 | 8.17 |
| Housebreaking | 99.89 | 22.11 | 37.17 | 16.82 | 32.64 | 100 | 79.72 | 78.06 |
| Socially Inappropriate Behavior with Dogs | 5.79 | 11.01 | 16.61 | 12.47 | 16.02 | 99.72 | 18.04 | 15.60 |
| Thunder Fear | 26.23 | 24.39 | 19.33 | 29.29 | 20.23 | 19.72 | 20.19 | 19.54 |
| Gait Moving Out | 100 | 100 | 100 | 100 | 100 | 100 | 100 | 100 |
| Avoids Car Exhaust | 100 | 100 | 100 | 100 | 100 | 100 | 100 | 100 |
| Kennels Poorly | 99.72 | 87.38 | 85.94 | 83.53 | 87.36 | 99.57 | 9.73 | 9.71 |
| Working Speed | 100 | 100 | 100 | 100 | 100 | 100 | 100 | 100 |

| | | | | | | | | |
|---|---|---|---|---|---|---|---|---|
| Innate Desire to Work | 100 | 100 | 100 | 100 | 100 | 100 | 100 | 100 |

***Supplemental Table 5.*** Exploratory factor analysis results from the puppy test. These factors, with the addition of a kinesthetic factor, were used for subsequent analyses.

| BCL Item | Resilience | Relationship | Arousal Activated | Distraction |
|---|---|---|---|---|
| Noise Fear | 0.614 | | | |
| Fear of Novel Objects | 0.545 | | | |
| Handler Attachment | 0.545 | | | |
| Passive Response to Stress | 0.616 | | | |
| Self Modulation | 0.664 | | | |
| Handler Dog Team | | 0.883 | | |
| Unwilling | | 0.727 | | |
| Excitable | | | 0.758 | |
| Movement Excites | | | 0.586 | |
| Active Response to Stress | | | 0.528 | |
| High Energy | | | 0.496 | |
| Social Manners | | | 0.485 | |
| Scavenges | | | | 0.737 |
| Olfactory | | | | 0.530 |
| SS loadings | 2.517 | 1.659 | 2.280 | 0.999 |
| Proportion Var | 0.076 | 0.050 | 0.069 | 0.030 |

| Cumulative Var | 0.076 | 0.196 | 0.145 | 0.258 |
|---|---|---|---|---|

***Supplemental Table 6.*** Confirmatory factor analysis results indicate a moderately good fit of the puppy test evaluation to all other evaluations.

| Evaluation | Compared to | CFI | TLI | RMSEA | RMSEA Lower | RMSEA Upper |
|---|---|---|---|---|---|---|
| P | P | 0.80 | 0.751 | 0.077 | 0.075 | 0.078 |
| P | W1 | 0.768 | 0.704 | 0.070 | 0.068 | 0.071 |
| P | W2 | 0.785 | 0.726 | 0.072 | 0.070 | 0.074 |
| P | W2b | 0.775 | 0.071 | 0.075 | 0.073 | 0.077 |
| P | W3 | 0.783 | 0.724 | 0.070 | 0.069 | 0.072 |
| P | IFT | 0.799 | 0.744 | 0.083 | 0.082 | 0.085 |
| P | PB | 0.807 | 0.754 | 0.086 | 0.085 | 0.088 |
| P | FB | 0.779 | 0.718 | 0.091 | 0.089 | 0.092 |

***Supplemental Table 7.*** Confirmatory factor analysis results in the validation cohort. Overall, the results indicate that the model is a good fit for the validation cohort.

| Evaluation | Compared to | CFI | TLI | RMSEA | RMSEA Lower | RMSEA Upper |
|---|---|---|---|---|---|---|
| P | PB | 0.947 | 0.928 | 0.037 | 0.025 | 0.048 |
| P | FB | 0.786 | 0.712 | 0.080 | 0.070 | 0.090 |

***Supplemental Table 9.*** Kappa statistics, PPVs, and NPVs for groupings derived from factor analysis.

| Factor | Evaluation 1 | Evaluation 2 | Kappa Mean | Kappa LS | PPV Mean | PPV LS | NPV Mean | NPV LS |
|---|---|---|---|---|---|---|---|---|
| Resilience | P | W1 | -0.0333 | 0.0309 | 0.9982 | NA | 0.0000 | NA |
| Resilience | P | W2 | 0.0000 | 0.0466 | NA | NA | NA | NA |
| Resilience | P | W2b | 0.0278 | 0.0350 | 0.9930 | NA | 0.0000 | NA |

| | | | | | | | | |
|---|---|---|---|---|---|---|---|---|
| Resilience | P | W3 | 0.0496 | 0.0319 | 0.9983 | NA | 0.0500 | NA |
| Resilience | P | IFT | 0.0144 | 0.0151 | 0.9890 | 0.9141 | 0.0118 | 0.0993 |
| Resilience | P | PB | -0.0550 | 0.0035 | 0.9802 | NA | 0.0000 | NA |
| Resilience | P | FB | -0.0261 | -0.0144 | 0.9679 | NA | 0.0200 | NA |
| Resilience | W1 | W2 | 0.7329 | 0.2427 | NA | 0.9605 | NA | 0.2286 |
| Resilience | W1 | W2b | 0.4324 | 0.2223 | 0.9954 | 0.9432 | 0.0000 | 0.1739 |
| Resilience | W1 | W3 | 0.7167 | 0.1504 | 0.9988 | 0.9617 | 0.0000 | 0.1250 |
| Resilience | W1 | IFT | 0.0720 | 0.0318 | 0.9856 | 0.9121 | 0.0000 | 0.0806 |
| Resilience | W1 | PB | 0.1538 | 0.0258 | 0.9821 | 0.8069 | 0.0000 | 0.2128 |
| Resilience | W1 | FB | -0.0761 | -0.0024 | 0.9672 | 0.7930 | 0.0000 | 0.2973 |
| Resilience | W2 | W3 | 0.8416 | 0.2387 | NA | 0.9609 | NA | 0.1463 |
| Resilience | W2 | IFT | 0.2037 | 0.0508 | 0.9856 | 0.9011 | 0.0000 | 0.2174 |
| Resilience | W2 | PB | 0.0455 | -0.0148 | 0.9785 | 0.7974 | 0.0000 | 0.2593 |
| Resilience | W2 | FB | 0.0687 | 0.0218 | 0.9792 | 0.7781 | 0.0000 | 0.3043 |
| Resilience | W2b | IFT | 0.0696 | 0.0513 | 0.9887 | 0.9333 | 0.0000 | 0.1489 |
| Resilience | W2b | PB | 7.40148683083437e-16 | 0.0367 | 0.9803 | 0.7983 | 0.0000 | 0.2821 |
| Resilience | W2b | FB | 0.0270 | 0.0407 | 0.9614 | 0.7813 | 0.0000 | 0.2333 |
| Resilience | W3 | IFT | 0.1767 | 0.0881 | 0.9849 | 0.9066 | 0.0000 | 0.2273 |
| Resilience | W3 | PB | 0.0998 | 0.0299 | NA | 0.7918 | NA | 0.2963 |
| Resilience | W3 | FB | 0.2045 | 0.0471 | NA | 0.7944 | NA | 0.4667 |

| | | | | | | | | |
|---|---|---|---|---|---|---|---|---|
| Resilience | IFT | PB | 0.0024 | 0.0383 | 0.9774 | 0.8109 | 0.1000 | 0.2589 |
| Resilience | IFT | FB | 0.0741 | 0.0075 | 0.9674 | 0.7845 | 0.1429 | 0.2941 |
| Resilience | PB | FB | 0.6664 | 0.3690 | 0.9698 | 0.8446 | 0.2143 | 0.6211 |
| Relationship | P | W1 | -0.0006 | 0.0115 | 0.9837 | 0.9467 | 0.0000 | 0.0000 |
| Relationship | P | W2 | 0.0051 | 0.0194 | 0.9895 | NA | 0.0000 | NA |
| Relationship | P | W2b | 0.0358 | -0.0131 | 0.9870 | 0.9701 | 0.0000 | 0.0909 |
| Relationship | P | W3 | 0.0373 | -0.0168 | 0.9846 | NA | 0.0000 | NA |
| Relationship | P | IFT | 0.0414 | 0.0250 | 0.9351 | 0.8918 | 0.1579 | 0.2000 |
| Relationship | P | PB | 0.0403 | 0.0222 | 0.9728 | 0.9298 | 0.0000 | 0.0526 |
| Relationship | P | FB | -0.0454 | 0.0068 | 0.9787 | 0.9519 | 0.0625 | 0.1765 |
| Relationship | W1 | W2 | 0.4673 | 0.3035 | 0.9889 | 0.9600 | 0.1111 | 0.1458 |
| Relationship | W1 | W2b | 0.5313 | 0.2738 | 0.9919 | 0.9748 | 0.0000 | 0.0238 |
| Relationship | W1 | W3 | 0.4671 | 0.2141 | 0.9894 | 0.9755 | 0.0000 | 0.0612 |
| Relationship | W1 | IFT | 0.2075 | 0.0861 | 0.9451 | 0.9026 | 0.1111 | 0.1786 |
| Relationship | W1 | PB | 0.1013 | 0.0535 | 0.9719 | 0.9237 | 0.0000 | 0.0923 |
| Relationship | W1 | FB | 0.1022 | 0.0561 | 0.9834 | 0.9482 | 0.0000 | 0.0588 |
| Relationship | W2 | W3 | 0.4791 | 0.3200 | 0.9905 | 0.9803 | 0.0000 | 0.1277 |
| Relationship | W2 | IFT | 0.2343 | 0.1115 | 0.9208 | 0.8860 | 0.3125 | 0.2222 |
| Relationship | W2 | PB | 0.1340 | 0.0787 | 0.9704 | 0.9247 | 0.0000 | 0.0851 |
| Relationship | W2 | FB | 0.0754 | 0.0353 | 0.9806 | 0.9375 | 0.1000 | 0.0857 |
| Relationship | W2b | IFT | 0.1212 | 0.0833 | 0.9448 | 0.8897 | 0.3333 | 0.2188 |
| Relationship | W2b | PB | 0.0026 | 0.0145 | 0.9745 | 0.9317 | 0.0625 | 0.1333 |

| | | | | | | | | |
|---|---|---|---|---|---|---|---|---|
| Relationship | W2b | FB | 0.0463 | -0.010<br>1 | 0.9795 | 0.9498 | 0.0000 | 0.0000 |
| Relationship | W3 | IFT | 0.1893 | 0.1077 | 0.9148 | 0.8817 | 0.3333 | 0.2222 |
| Relationship | W3 | PB | 0.1747 | 0.0912 | 0.9675 | 0.9189 | 0.1538 | 0.1724 |
| Relationship | W3 | FB | 0.1127 | 0.0671 | 0.9804 | 0.9432 | 0.0000 | 0.0000 |
| Relationship | IFT | PB | 0.1590 | 0.0817 | 0.9749 | 0.9280 | 0.0902 | 0.1016 |
| Relationship | IFT | FB | 0.0997 | 0.0485 | 0.9808 | 0.9452 | 0.0642 | 0.0728 |
| Relationship | PB | FB | 0.6483 | 0.4316 | 0.9829 | 0.9597 | 0.3462 | 0.4333 |
| Arousal Activated | P | W1 | 0.5183 | 0.0974 | 0.9845 | 0.9309 | 0.0909 | 0.1124 |
| Arousal Activated | P | W2 | -0.0125 | 0.0369 | 0.9825 | 0.8864 | 0.1000 | 0.0727 |
| Arousal Activated | P | W2b | 0.2091 | 0.1089 | 0.9753 | 0.9253 | 0.1000 | 0.1964 |
| Arousal Activated | P | W3 | -0.0189 | 0.0620 | 0.9828 | 0.8916 | 0.1000 | 0.1034 |
| Arousal Activated | P | IFT | -0.0577 | 0.0374 | 0.9817 | 0.8957 | 0.0667 | 0.1600 |
| Arousal Activated | P | PB | -0.0171 | 0.0091 | 0.9553 | 0.8546 | 0.0000 | 0.1364 |
| Arousal Activated | P | FB | 0.0324 | 0.0470 | 0.9668 | 0.8561 | 0.0000 | 0.1549 |
| Arousal Activated | W1 | W2 | 0.7811 | 0.3477 | 0.9878 | 0.9397 | 0.1429 | 0.3158 |
| Arousal Activated | W1 | W2b | 0.7392 | 0.3319 | 0.9802 | 0.9354 | 0.1333 | 0.3704 |
| Arousal Activated | W1 | W3 | 0.7927 | 0.2604 | 0.9906 | 0.9297 | 0.1000 | 0.2113 |
| Arousal Activated | W1 | IFT | 0.0670 | 0.0746 | 0.9821 | 0.8972 | 0.0645 | 0.1389 |

| | | | | | | | | |
|---|---|---|---|---|---|---|---|---|
| Arousal Activated | W1 | PB | 0.1282 | 0.0818 | 0.9620 | 0.8539 | 0.0909 | 0.2262 |
| Arousal Activated | W1 | FB | 0.1389 | 0.0267 | 0.9709 | 0.8429 | 0.1111 | 0.1467 |
| Arousal Activated | W2 | W3 | 0.7677 | 0.3787 | 0.9905 | 0.9387 | 0.1538 | 0.3467 |
| Arousal Activated | W2 | IFT | 0.0954 | 0.0843 | 0.9770 | 0.8969 | 0.0588 | 0.1566 |
| Arousal Activated | W2 | PB | 0.0984 | 0.1032 | 0.9587 | 0.8479 | 0.0000 | 0.1714 |
| Arousal Activated | W2 | FB | 0.1273 | 0.0984 | 0.9771 | 0.8514 | 0.0000 | 0.1636 |
| Arousal Activated | W2b | IFT | 0.1911 | 0.0862 | 0.9842 | 0.8943 | 0.0417 | 0.1818 |
| Arousal Activated | W2b | PB | 0.1348 | 0.1057 | 0.9582 | 0.8500 | 0.1364 | 0.2969 |
| Arousal Activated | W2b | FB | 0.0018 | 0.0636 | 0.9588 | 0.8535 | 0.1333 | 0.2549 |
| Arousal Activated | W3 | IFT | 0.1767 | 0.1284 | 0.9752 | 0.8987 | 0.0000 | 0.2340 |
| Arousal Activated | W3 | PB | 0.0791 | 0.0923 | 0.9609 | 0.8483 | 0.1000 | 0.1951 |
| Arousal Activated | W3 | FB | 0.0164 | 0.0629 | 0.9651 | 0.8487 | 0.0000 | 0.1719 |
| Arousal Activated | IFT | PB | 0.2084 | 0.0734 | 0.9636 | 0.8578 | 0.1778 | 0.2211 |
| Arousal Activated | IFT | FB | 0.2289 | 0.0824 | 0.9605 | 0.8544 | 0.0938 | 0.2138 |
| Arousal Activated | PB | FB | 0.7556 | 0.4478 | 0.9703 | 0.9030 | 0.3673 | 0.5510 |
| Distraction | P | W1 | 0.0047 | 0.0312 | 0.9759 | 0.9485 | 0.0714 | 0.0690 |
| Distraction | P | W2 | -0.0393 | 0.0037 | 0.9629 | 0.9209 | 0.0625 | 0.1154 |

| | | | | | | | | |
|---|---|---|---|---|---|---|---|---|
| Distraction | P | W2b | -0.0318 | -0.0561 | 0.9791 | 0.9472 | 0.0833 | 0.0455 |
| Distraction | P | W3 | -0.0753 | 0.0014 | 0.9602 | 0.9221 | 0.0000 | 0.0000 |
| Distraction | P | IFT | 0.0661 | 0.0112 | 0.9790 | 0.9566 | 0.0952 | 0.1190 |
| Distraction | P | PB | -0.0108 | 0.0302 | 0.9558 | 0.9102 | 0.0417 | 0.1000 |
| Distraction | P | FB | 0.0285 | 0.0205 | 0.9691 | 0.9401 | 0.0500 | 0.0333 |
| Distraction | W1 | W2 | 0.3518 | 0.2370 | 0.9697 | 0.9397 | 0.1379 | 0.2241 |
| Distraction | W1 | W2b | 0.2164 | 0.1649 | 0.9837 | 0.9546 | 0.0625 | 0.1351 |
| Distraction | W1 | W3 | 0.3084 | 0.2101 | 0.9774 | 0.9485 | 0.0741 | 0.1569 |
| Distraction | W1 | IFT | 0.0231 | 0.0284 | 0.9768 | 0.9570 | 0.0000 | 0.0789 |
| Distraction | W1 | PB | 0.0846 | 0.0413 | 0.9560 | 0.9162 | 0.1071 | 0.1290 |
| Distraction | W1 | FB | 0.0781 | 0.0230 | 0.9621 | 0.9348 | 0.0000 | 0.1034 |
| Distraction | W2 | W3 | 0.3604 | 0.2756 | 0.9807 | 0.9600 | 0.1613 | 0.1724 |
| Distraction | W2 | IFT | -0.0383 | 0.0055 | 0.9646 | 0.9546 | 0.0303 | 0.0645 |
| Distraction | W2 | PB | 0.0576 | 0.0933 | 0.9596 | 0.9314 | 0.1304 | 0.2553 |
| Distraction | W2 | FB | 0.0399 | 0.0738 | 0.9687 | 0.9507 | 0.0500 | 0.1282 |
| Distraction | W2b | IFT | 0.0713 | 0.0351 | 0.9770 | 0.9470 | 0.0000 | 0.0208 |
| Distraction | W2b | PB | 0.0901 | 0.0097 | 0.9521 | 0.8996 | 0.0000 | 0.0556 |
| Distraction | W2b | FB | 0.0089 | 0.0555 | 0.9540 | 0.9184 | 0.1000 | 0.1212 |
| Distraction | W3 | IFT | 0.0910 | 0.0515 | 0.9664 | 0.9537 | 0.0000 | 0.0533 |
| Distraction | W3 | PB | 0.0586 | 0.0790 | 0.9496 | 0.9105 | 0.0968 | 0.1364 |
| Distraction | W3 | FB | 0.1142 | 0.0508 | 0.9600 | 0.9418 | 0.0400 | 0.0727 |
| Distraction | IFT | PB | 0.0229 | 0.0225 | 0.9553 | 0.9077 | 0.0610 | 0.1032 |
| Distraction | IFT | FB | 0.0073 | 0.0273 | 0.9599 | 0.9341 | 0.0758 | 0.1176 |

| | | | | | | | | |
|---|---|---|---|---|---|---|---|---|
| Distraction | PB | FB | 0.5939 | 0.4345 | 0.9754 | 0.9627 | 0.4085 | 0.4041 |
| Kinesthetic | P | W1 | 0.1944 | 0.0516 | 0.9931 | 0.9742 | 0.0000 | 0.0400 |
| Kinesthetic | P | W2 | -0.0125 | -0.0138 | 0.9876 | 0.9554 | 0.0000 | 0.0303 |
| Kinesthetic | P | W2b | 0.0394 | 0.0616 | 0.9908 | 0.9527 | 0.0000 | 0.1000 |
| Kinesthetic | P | W3 | 0.0029 | -0.0105 | 0.9948 | 0.9580 | 0.0000 | 0.0313 |
| Kinesthetic | P | IFT | 0.0462 | 0.0237 | 0.9839 | 0.9234 | 0.0000 | 0.0847 |
| Kinesthetic | P | PB | -0.0046 | -0.0061 | 0.9556 | 0.8589 | 0.0000 | 0.0851 |
| Kinesthetic | P | FB | 0.0842 | 0.0193 | 0.9744 | 0.9031 | 0.0000 | 0.0270 |
| Kinesthetic | W1 | W2 | 0.3567 | 0.1981 | 0.9913 | 0.9644 | 0.0000 | 0.2000 |
| Kinesthetic | W1 | W2b | 0.3101 | 0.1760 | 0.9896 | 0.9538 | 0.1429 | 0.1786 |
| Kinesthetic | W1 | W3 | 0.2178 | 0.1301 | 0.9953 | 0.9669 | 0.0000 | 0.1818 |
| Kinesthetic | W1 | IFT | 0.0668 | 0.0729 | 0.9817 | 0.9198 | 0.0000 | 0.2000 |
| Kinesthetic | W1 | PB | -0.0369 | -0.0007 | 0.9608 | 0.8631 | 0.0000 | 0.1481 |
| Kinesthetic | W1 | FB | 0.0194 | 0.0175 | 0.9773 | 0.9065 | 0.0000 | 0.1200 |
| Kinesthetic | W2 | W3 | 0.4402 | 0.2991 | 0.9965 | 0.9674 | 0.0000 | 0.1667 |
| Kinesthetic | W2 | IFT | 0.1254 | 0.0923 | 0.9834 | 0.9209 | 0.0000 | 0.2703 |
| Kinesthetic | W2 | PB | 0.0101 | 0.0530 | 0.9641 | 0.8677 | 0.0000 | 0.2727 |
| Kinesthetic | W2 | FB | 0.0279 | 0.0756 | 0.9806 | 0.9197 | 0.1429 | 0.1852 |
| Kinesthetic | W2b | IFT | 0.0686 | 0.0992 | 0.9855 | 0.9350 | 0.1250 | 0.3143 |
| Kinesthetic | W2b | PB | 0.0228 | 0.0838 | 0.9615 | 0.8874 | 0.1111 | 0.3143 |
| Kinesthetic | W2b | FB | 0.1950 | 0.1329 | 0.9729 | 0.9155 | 0.1429 | 0.2069 |

| | | | | | | | | |
|---|---|---|---|---|---|---|---|---|
| Kinesthetic | W3 | IFT | -0.0083 | 0.0837 | 0.9829 | 0.9207 | 0.2000 | 0.1765 |
| Kinesthetic | W3 | PB | 0.0082 | 0.0184 | 0.9645 | 0.8673 | 0.0000 | 0.2258 |
| Kinesthetic | W3 | FB | 0.0641 | 0.0311 | 0.9806 | 0.9158 | 0.0000 | 0.0870 |
| Kinesthetic | IFT | PB | 0.1450 | 0.1808 | 0.9637 | 0.8900 | 0.3333 | 0.4000 |
| Kinesthetic | IFT | FB | 0.1540 | 0.1418 | 0.9757 | 0.9249 | 0.1000 | 0.2895 |
| Kinesthetic | PB | FB | 0.6747 | 0.5839 | 0.9873 | 0.9627 | 0.5000 | 0.5503 |

***Supplementary Table 10.*** Kappa statistics, PPVs, and NPVs for groupings derived from trainer-determined release reasons.

| Release Reason | Evaluation 1 | Evaluation 2 | Kappa Mean | Kappa LS | PPV Mean | PPV LS | NPV Mean | NPV LS |
|---|---|---|---|---|---|---|---|---|
| Adaptability | P | W1 | 0.0151 | 0.0471 | 0.9991 | NA | 0.0000 | NA |
| Adaptability | P | W2 | 0.0192 | 0.0487 | NA | NA | NA | NA |
| Adaptability | P | W2b | 0.0675 | 0.0314 | 0.9934 | 0.9822 | 0.0000 | 0.0000 |
| Adaptability | P | W3 | -0.0165 | 0.0423 | NA | NA | NA | NA |
| Adaptability | P | IFT | -0.0133 | -0.0561 | NA | NA | NA | NA |
| Adaptability | P | PB | -0.0323 | -0.0327 | 0.9800 | 0.8939 | 0.0000 | 0.1053 |
| Adaptability | P | FB | -0.0222 | -0.0341 | 0.9839 | 0.8531 | 0.0000 | 0.1042 |
| Adaptability | W1 | W2 | 0.3409 | 0.2418 | NA | NA | NA | NA |
| Adaptability | W1 | W2b | 0.2669 | 0.2030 | NA | NA | NA | NA |
| Adaptability | W1 | W3 | 0.1936 | 0.1800 | NA | NA | NA | NA |

| | | | | | | | | | |
|---|---|---|---|---|---|---|---|---|---|
| Adaptability | W1 | IFT | -0.0699 | -0.0753 | NA | NA | NA | NA |
| Adaptability | W1 | PB | 0.0782 | 0.0150 | NA | NA | NA | NA |
| Adaptability | W1 | FB | 0.0376 | 0.0282 | NA | NA | NA | NA |
| Adaptability | W2 | W3 | 0.3173 | 0.3114 | 0.9977 | NA | 0.0000 | NA |
| Adaptability | W2 | IFT | -0.0532 | -0.0725 | NA | NA | NA | NA |
| Adaptability | W2 | PB | 0.0892 | 0.0571 | 0.9899 | NA | 0.0000 | NA |
| Adaptability | W2 | FB | 0.1504 | 0.0612 | 0.9824 | NA | 0.0000 | NA |
| Adaptability | W2b | IFT | -0.0825 | -0.1265 | NA | NA | NA | NA |
| Adaptability | W2b | PB | 0.1461 | 0.0677 | 0.9816 | NA | 0.3333 | NA |
| Adaptability | W2b | FB | 0.1692 | 0.0329 | 0.9730 | NA | 0.3333 | NA |
| Adaptability | W3 | IFT | -0.0680 | -0.0965 | NA | NA | NA | NA |
| Adaptability | W3 | PB | 0.0677 | 0.0545 | 0.9867 | NA | 0.0000 | NA |
| Adaptability | W3 | FB | 0.1660 | 0.0647 | 0.9778 | NA | 0.0000 | NA |
| Adaptability | IFT | PB | -0.1108 | -0.0064 | NA | NA | NA | NA |
| Adaptability | IFT | FB | 0.0704 | 0.0410 | NA | NA | NA | NA |
| Adaptability | PB | FB | 0.6881 | 0.5232 | 0.9815 | 0.9136 | 0.5294 | 0.7328 |

| | | | | | | | | |
|---|---|---|---|---|---|---|---|---|
| Chasing | P | W1 | 0.0941 | 0.0922 | 0.9895 | 0.9834 | 0.0370 | 0.0357 |
| Chasing | P | W2 | -0.0212 | -0.0051 | 0.9946 | NA | 0.0000 | NA |
| Chasing | P | W2b | 0.0863 | 0.0956 | 0.9881 | 0.9814 | 0.0435 | 0.0417 |
| Chasing | P | W3 | -0.0334 | -0.0110 | 0.9930 | 0.9789 | 0.0417 | 0.0417 |
| Chasing | P | IFT | 0.0526 | 0.0465 | 0.9674 | 0.9657 | 0.1053 | 0.1026 |
| Chasing | P | PB | -0.0002 | 0.0013 | 0.9553 | 0.9414 | 0.0606 | 0.0882 |
| Chasing | P | FB | 0.0063 | 0.0125 | 0.9688 | 0.9524 | 0.0345 | 0.0667 |
| Chasing | W1 | W2 | 0.2625 | 0.2041 | 0.9978 | NA | 0.0000 | NA |
| Chasing | W1 | W2b | 0.3038 | 0.2108 | 0.9884 | 0.9848 | 0.1000 | 0.2000 |
| Chasing | W1 | W3 | 0.3143 | 0.1631 | 0.9918 | 0.9858 | 0.0000 | 0.0500 |
| Chasing | W1 | IFT | 0.0684 | 0.0668 | 0.9701 | 0.9686 | 0.0000 | 0.0690 |
| Chasing | W1 | PB | 0.0578 | 0.0474 | 0.9481 | 0.9326 | 0.0667 | 0.1154 |
| Chasing | W1 | FB | 0.0602 | 0.0562 | 0.9678 | 0.9517 | 0.0000 | 0.0435 |
| Chasing | W2 | W3 | 0.4001 | 0.2899 | 0.9918 | NA | 0.0000 | NA |
| Chasing | W2 | IFT | 0.0345 | 0.0339 | 0.9749 | 0.9727 | 0.0000 | 0.0000 |

| | | | | | | | | |
|---|---|---|---|---|---|---|---|---|
| Chasing | W2 | PB | 0.0300 | 0.0444 | 0.9540 | 0.9422 | 0.1250 | 0.0833 |
| Chasing | W2 | FB | 0.0415 | 0.0720 | 0.9661 | NA | 0.0000 | NA |
| Chasing | W2b | IFT | 0.0412 | 0.0431 | 0.9627 | 0.9603 | 0.1000 | 0.0556 |
| Chasing | W2b | PB | 0.0662 | 0.0800 | 0.9442 | 0.9249 | 0.3333 | 0.2500 |
| Chasing | W2b | FB | -0.0035 | 0.0380 | 0.9627 | 0.9417 | 0.1667 | 0.2308 |
| Chasing | W3 | IFT | 0.0542 | 0.0485 | 0.9722 | 0.9710 | 0.0000 | 0.0556 |
| Chasing | W3 | PB | 0.0254 | 0.0561 | 0.9452 | 0.9276 | 0.0000 | 0.0526 |
| Chasing | W3 | FB | 0.0474 | 0.0785 | 0.9680 | 0.9506 | 0.0000 | 0.1333 |
| Chasing | IFT | PB | 0.0232 | 0.0232 | 0.9458 | 0.9302 | 0.0896 | 0.0972 |
| Chasing | IFT | FB | 0.0252 | 0.0083 | 0.9594 | 0.9412 | 0.0806 | 0.0923 |
| Chasing | PB | FB | 0.4551 | 0.3845 | 0.9738 | 0.9645 | 0.3333 | 0.3750 |
| Emotional Composure | P | W1 | 0.1773 | 0.0651 | 0.9888 | 0.9434 | 0.0000 | 0.0588 |
| Emotional Composure | P | W2 | 0.1923 | 0.0756 | 0.9828 | 0.9130 | 0.0000 | 0.0536 |
| Emotional Composure | P | W2b | -0.0715 | 0.0482 | 0.9857 | 0.9386 | 0.0000 | 0.0875 |
| Emotional Composure | P | W3 | 0.0128 | 0.0475 | 0.9813 | 0.9403 | 0.0000 | 0.0280 |

| | | | | | | | | |
|---|---|---|---|---|---|---|---|---|
| Emotional Composure | P | IFT | -0.0320 | 0.0069 | 0.9936 | 0.9061 | 0.0000 | 0.1158 |
| Emotional Composure | P | PB | 0.1256 | 0.0010 | 0.9315 | 0.7668 | 0.0000 | 0.2150 |
| Emotional Composure | P | FB | 0.2733 | -0.0106 | 0.9232 | 0.7695 | 0.1000 | 0.1914 |
| Emotional Composure | W1 | W2 | 0.5802 | 0.3174 | 0.9912 | 0.9549 | 0.2500 | 0.3333 |
| Emotional Composure | W1 | W2b | 0.2776 | 0.2187 | 0.9861 | 0.9400 | 0.2222 | 0.2439 |
| Emotional Composure | W1 | W3 | 0.4847 | 0.1871 | 0.9895 | 0.9523 | 0.0909 | 0.1600 |
| Emotional Composure | W1 | IFT | 0.1960 | 0.0334 | 0.9899 | 0.9013 | 0.0714 | 0.1299 |
| Emotional Composure | W1 | PB | 0.0381 | 0.0496 | 0.9321 | 0.7694 | 0.0000 | 0.3684 |
| Emotional Composure | W1 | FB | 0.0668 | 0.0353 | 0.9237 | 0.7548 | 0.0000 | 0.2917 |
| Emotional Composure | W2 | W3 | 0.6816 | 0.3239 | 0.9894 | 0.9589 | 0.0909 | 0.1786 |
| Emotional Composure | W2 | IFT | -0.0093 | 0.0468 | 0.9844 | 0.9016 | 0.0000 | 0.2623 |
| Emotional Composure | W2 | PB | 0.1947 | 0.0629 | 0.9336 | 0.7574 | 0.0000 | 0.2222 |
| Emotional Composure | W2 | FB | 0.1461 | 0.0471 | 0.9290 | 0.7538 | 0.2857 | 0.3684 |
| Emotional Composure | W2b | IFT | -0.0614 | -0.0122 | 0.9917 | 0.9108 | 0.0000 | 0.1034 |
| Emotional Composure | W2b | PB | 0.1343 | 0.0508 | 0.9289 | 0.7577 | 0.0000 | 0.3400 |

| | | | | | | | | |
|---|---|---|---|---|---|---|---|---|
| Emotional Composure | W2b | FB | 0.0282 | 0.0179 | 0.9091 | 0.7522 | 0.0000 | 0.2222 |
| Emotional Composure | W3 | IFT | 0.2094 | 0.1130 | 0.9857 | 0.9059 | 0.0909 | 0.2800 |
| Emotional Composure | W3 | PB | 0.0824 | 0.0399 | 0.9343 | 0.7500 | 0.0000 | 0.3333 |
| Emotional Composure | W3 | FB | -0.0574 | 0.0528 | 0.9221 | 0.7603 | 0.0000 | 0.3750 |
| Emotional Composure | IFT | PB | 0.0758 | 0.0408 | 0.9366 | 0.7795 | 0.1667 | 0.2770 |
| Emotional Composure | IFT | FB | 0.0902 | 0.0472 | 0.9160 | 0.7599 | 0.0000 | 0.4182 |
| Emotional Composure | PB | FB | 0.7015 | 0.3981 | 0.9292 | 0.8261 | 0.4200 | 0.6173 |
| Distraction | P | W1 | 0.1189 | 0.0590 | 0.9758 | 0.9555 | 0.0000 | 0.0392 |
| Distraction | P | W2 | -0.0158 | 0.0073 | 0.9104 | 0.8738 | 0.0769 | 0.1860 |
| Distraction | P | W2b | -0.0298 | -0.0281 | 0.9332 | 0.8977 | 0.0000 | 0.0652 |
| Distraction | P | W3 | -0.0315 | -0.0026 | 0.9201 | 0.8834 | 0.0588 | 0.1364 |
| Distraction | P | IFT | 0.0129 | 0.0152 | 0.9759 | 0.9353 | 0.1250 | 0.1538 |
| Distraction | P | PB | 0.0071 | 0.0342 | 0.9251 | 0.8698 | 0.1111 | 0.1449 |
| Distraction | P | FB | -0.0159 | 0.0036 | 0.9205 | 0.8858 | 0.0833 | 0.0702 |
| Distraction | W1 | W2 | 0.3682 | 0.2865 | 0.9443 | 0.9223 | 0.3043 | 0.3696 |

| | | | | | | | | |
|---|---|---|---|---|---|---|---|---|
| Distraction | W1 | W2b | 0.2159 | 0.2171 | 0.9508 | 0.9185 | 0.1500 | 0.2963 |
| Distraction | W1 | W3 | 0.3297 | 0.2420 | 0.9457 | 0.9274 | 0.1500 | 0.2927 |
| Distraction | W1 | IFT | 0.0637 | 0.0535 | 0.9777 | 0.9305 | 0.0000 | 0.1455 |
| Distraction | W1 | PB | 0.0721 | 0.0730 | 0.9231 | 0.8689 | 0.0909 | 0.2143 |
| Distraction | W1 | FB | 0.0098 | 0.0585 | 0.9208 | 0.8842 | 0.0556 | 0.1081 |
| Distraction | W2 | W3 | 0.4881 | 0.3520 | 0.9626 | 0.9471 | 0.2143 | 0.2892 |
| Distraction | W2 | IFT | 0.0704 | 0.0533 | 0.9801 | 0.9206 | 0.0286 | 0.1333 |
| Distraction | W2 | PB | 0.1035 | 0.1318 | 0.9354 | 0.8895 | 0.1458 | 0.2368 |
| Distraction | W2 | FB | 0.1320 | 0.0855 | 0.9368 | 0.9115 | 0.1190 | 0.1935 |
| Distraction | W2b | IFT | 0.0497 | 0.0595 | 0.9740 | 0.9207 | 0.0196 | 0.0659 |
| Distraction | W2b | PB | 0.0456 | 0.0280 | 0.9204 | 0.8603 | 0.1087 | 0.1333 |
| Distraction | W2b | FB | 0.0011 | 0.0433 | 0.9119 | 0.8752 | 0.1250 | 0.1864 |
| Distraction | W3 | IFT | 0.1091 | 0.0870 | 0.9756 | 0.9168 | 0.0811 | 0.1273 |
| Distraction | W3 | PB | 0.1089 | 0.1053 | 0.9283 | 0.8738 | 0.1029 | 0.2268 |
| Distraction | W3 | FB | 0.0567 | 0.0826 | 0.9297 | 0.8940 | 0.0357 | 0.1081 |

| | | | | | | | | |
|---|---|---|---|---|---|---|---|---|
| Distraction | IFT | PB | 0.0164 | 0.0380 | 0.9180 | 0.8647 | 0.1294 | 0.1598 |
| Distraction | IFT | FB | 0.0399 | 0.0425 | 0.9177 | 0.8927 | 0.1528 | 0.2298 |
| Distraction | PB | FB | 0.5003 | 0.4377 | 0.9503 | 0.9349 | 0.5122 | 0.4829 |
| Dog Problems | P | W1 | -0.0050 | 0.0318 | NA | NA | NA | NA |
| Dog Problems | P | W2 | -0.0115 | 0.0512 | NA | NA | NA | NA |
| Dog Problems | P | W2b | -0.0047 | 0.0182 | NA | NA | NA | NA |
| Dog Problems | P | W3 | -0.0105 | -0.0001 | NA | NA | NA | NA |
| Dog Problems | P | IFT | -0.0044 | 0.0015 | NA | NA | NA | NA |
| Dog Problems | P | PB | 0.0485 | -0.0092 | NA | NA | NA | NA |
| Dog Problems | P | FB | -0.0107 | 0.0514 | NA | NA | NA | NA |
| Dog Problems | W1 | W2 | -0.0020 | 0.2338 | NA | NA | NA | NA |
| Dog Problems | W1 | W2b | -0.0017 | 0.1240 | NA | NA | NA | NA |
| Dog Problems | W1 | W3 | -0.0045 | 0.1426 | NA | NA | NA | NA |
| Dog Problems | W1 | IFT | -0.0031 | 0.0589 | NA | NA | NA | NA |
| Dog Problems | W1 | PB | -0.0033 | 0.0075 | NA | NA | NA | NA |
| Dog Problems | W1 | FB | -0.0033 | -0.0090 | NA | NA | NA | NA |
| Dog Problems | W2 | W3 | 0.4972 | 0.3449 | NA | NA | NA | NA |
| Dog Problems | W2 | IFT | 0.2199 | 0.0875 | NA | NA | NA | NA |
| Dog Problems | W2 | PB | -0.0028 | -0.0351 | NA | NA | NA | NA |

| | | | | | | | | |
|---|---|---|---|---|---|---|---|---|
| Dog Problems | W2 | FB | -0.0018 | 0.0004 | NA | NA | NA | NA |
| Dog Problems | W2b | IFT | 0.0000 | 0.1068 | NA | NA | NA | NA |
| Dog Problems | W2b | PB | -0.0026 | 0.0255 | NA | NA | NA | NA |
| Dog Problems | W2b | FB | -0.0022 | 0.0127 | NA | NA | NA | NA |
| Dog Problems | W3 | IFT | 0.1974 | 0.0838 | NA | NA | NA | NA |
| Dog Problems | W3 | PB | 0.0554 | 0.0364 | NA | NA | NA | NA |
| Dog Problems | W3 | FB | -0.0021 | 0.0509 | NA | NA | NA | NA |
| Dog Problems | IFT | PB | -0.0014 | 0.0097 | NA | NA | NA | NA |
| Dog Problems | IFT | FB | -0.0019 | 0.0217 | NA | NA | NA | NA |
| Dog Problems | PB | FB | 0.2357 | 0.3801 | 0.9988 | NA | 1.0000 | NA |
| Environmental Soundness | P | W1 | -0.0322 | 0.0155 | NA | 0.9690 | NA | 0.0146 |
| Environmental Soundness | P | W2 | 0.0000 | 0.0211 | NA | 0.9796 | NA | 0.0215 |
| Environmental Soundness | P | W2b | 0.0066 | 0.0058 | NA | 0.9621 | NA | 0.0274 |
| Environmental Soundness | P | W3 | 0.0073 | 0.0209 | NA | NA | NA | NA |
| Environmental Soundness | P | IFT | -0.0205 | 0.0249 | 0.9945 | 0.8934 | 0.0000 | 0.1213 |
| Environmental Soundness | P | PB | -0.0724 | 0.0083 | 0.9979 | 0.8975 | 0.0000 | 0.1135 |
| Environmental Soundness | P | FB | 0.0036 | -0.0453 | 0.9974 | 0.9142 | 0.0000 | 0.0473 |
| Environmental Soundness | W1 | W2 | 0.6585 | 0.1862 | NA | 0.9745 | NA | 0.0476 |

| | | | | | | | | |
|---|---|---|---|---|---|---|---|---|
| Environmental Soundness | W1 | IFT | 0.0321 | 0.0395 | NA | 0.9005 | NA | 0.0750 |
| Environmental Soundness | W1 | PB | -0.0182 | 0.0141 | NA | 0.9117 | NA | 0.0938 |
| Environmental Soundness | W1 | FB | 0.0000 | -0.0126 | NA | 0.9307 | NA | 0.0385 |
| Environmental Soundness | W2 | W3 | -0.0067 | 0.2561 | NA | 0.9702 | NA | 0.1905 |
| Environmental Soundness | W2 | IFT | 0.1670 | 0.0760 | NA | 0.8818 | NA | 0.1500 |
| Environmental Soundness | W2 | PB | 0.1351 | 0.0226 | NA | 0.8982 | NA | 0.1875 |
| Environmental Soundness | W2 | FB | 6.55031584528 84e-15 | -0.0135 | NA | 0.9380 | NA | 0.0000 |
| Environmental Soundness | W2b | IFT | 0.0000 | 0.0306 | NA | 0.9142 | NA | 0.1429 |
| Environmental Soundness | W2b | PB | 0.1254 | 0.0300 | NA | 0.9090 | NA | 0.1875 |
| Environmental Soundness | W2b | FB | 0.0000 | -0.0067 | NA | NA | NA | NA |
| Environmental Soundness | W3 | IFT | -0.0186 | 0.0912 | NA | 0.8793 | NA | 0.3200 |
| Environmental Soundness | W3 | PB | 0.0000 | 0.0375 | NA | 0.9019 | NA | 0.1111 |
| Environmental Soundness | W3 | FB | 0.1483 | 0.0532 | NA | 0.9340 | NA | 0.0833 |
| Environmental Soundness | IFT | PB | 0.0542 | 0.0533 | 0.9934 | 0.9081 | 0.0000 | 0.1333 |

| | | | | | | | | |
|---|---|---|---|---|---|---|---|---|
| Environmental Soundness | IFT | FB | 0.0688 | 0.0425 | 0.9979 | 0.9300 | 0.0000 | 0.1127 |
| Environmental Soundness | PB | FB | 0.6538 | 0.3903 | 0.9976 | 0.9422 | 0.3333 | 0.3426 |
| Environmental Soundness | W1 | W2b | NA | 0.2259 | NA | 0.9680 | NA | 0.0690 |
| Environmental Soundness | W1 | W3 | NA | 0.1418 | NA | 0.9753 | NA | 0.1053 |
| Fear of Heights | P | W1 | 0.0665 | 0.0665 | NA | NA | NA | NA |
| Fear of Heights | P | W2 | 0.0322 | 0.0322 | NA | NA | NA | NA |
| Fear of Heights | P | W2b | 0.0421 | 0.0421 | NA | NA | NA | NA |
| Fear of Heights | P | W3 | 0.0428 | 0.0428 | NA | NA | NA | NA |
| Fear of Heights | P | IFT | -0.1418 | -0.1418 | NA | NA | NA | NA |
| Fear of Heights | P | PB | 0.0282 | 0.0282 | NA | NA | NA | NA |
| Fear of Heights | P | FB | 0.0284 | 0.0284 | NA | NA | NA | NA |
| Fear of Heights | W1 | W2 | 0.2563 | 0.2563 | 0.9754 | 0.9754 | 0.1875 | 0.1875 |
| Fear of Heights | W1 | W2b | 0.1678 | 0.1678 | NA | NA | NA | NA |
| Fear of Heights | W1 | W3 | 0.1465 | 0.1465 | NA | NA | NA | NA |
| Fear of Heights | W1 | IFT | 0.3250 | 0.3250 | NA | NA | NA | NA |
| Fear of Heights | W1 | PB | 0.0769 | 0.0769 | 0.9727 | 0.9727 | 0.0000 | 0.0000 |

| | | | | | | | | |
|---|---|---|---|---|---|---|---|---|
| Fear of Heights | W1 | FB | 0.0632 | 0.0632 | NA | NA | NA | NA |
| Fear of Heights | W2 | W3 | 0.2578 | 0.2578 | NA | NA | NA | NA |
| Fear of Heights | W2 | IFT | 0.3419 | 0.3419 | NA | NA | NA | NA |
| Fear of Heights | W2 | PB | 0.1031 | 0.1031 | 0.9696 | 0.9696 | 0.2222 | 0.2222 |
| Fear of Heights | W2 | FB | 0.0431 | 0.0431 | NA | NA | NA | NA |
| Fear of Heights | W2b | IFT | 0.3774 | 0.3774 | NA | NA | NA | NA |
| Fear of Heights | W2b | PB | 0.1176 | 0.1176 | NA | NA | NA | NA |
| Fear of Heights | W2b | FB | 0.0518 | 0.0518 | NA | NA | NA | NA |
| Fear of Heights | W3 | IFT | 0.2953 | 0.2953 | NA | NA | NA | NA |
| Fear of Heights | W3 | PB | 0.0787 | 0.0787 | NA | NA | NA | NA |
| Fear of Heights | W3 | FB | 0.0418 | 0.0418 | NA | NA | NA | NA |
| Fear of Heights | IFT | PB | 0.3043 | 0.3043 | NA | NA | NA | NA |
| Fear of Heights | IFT | FB | -0.0465 | -0.0465 | NA | NA | NA | NA |
| Fear of Heights | PB | FB | 0.4246 | 0.4246 | 0.9879 | 0.9879 | 0.1667 | 0.1667 |
| Manners | P | W1 | 0.0548 | 0.0452 | 0.9905 | 0.9472 | 0.0000 | 0.0769 |
| Manners | P | W2 | 0.0899 | 0.0740 | NA | NA | NA | NA |

| | | | | | | | | |
|---|---|---|---|---|---|---|---|---|
| Manners | P | W2b | -0.0317 | 0.0100 | 0.9895 | 0.9397 | 0.0000 | 0.0769 |
| Manners | P | W3 | 0.0594 | 0.0444 | NA | NA | NA | NA |
| Manners | P | IFT | 0.0054 | 0.0255 | 0.9879 | 0.9401 | 0.0476 | 0.0952 |
| Manners | P | PB | 0.0185 | 0.0116 | 0.9862 | 0.9640 | 0.0000 | 0.0000 |
| Manners | P | FB | 0.0020 | 0.0162 | 0.9865 | 0.9704 | 0.0000 | 0.0000 |
| Manners | W1 | W2 | 0.4563 | 0.3588 | 0.9900 | 0.9430 | 0.0714 | 0.3448 |
| Manners | W1 | W2b | 0.4285 | 0.2982 | 0.9954 | 0.9469 | 0.4286 | 0.3556 |
| Manners | W1 | W3 | 0.3689 | 0.2365 | 0.9929 | 0.9514 | 0.0000 | 0.1475 |
| Manners | W1 | IFT | 0.0592 | 0.0458 | 0.9860 | 0.9409 | 0.0000 | 0.0380 |
| Manners | W1 | PB | 0.0188 | 0.0094 | 0.9868 | 0.9683 | 0.0000 | 0.0175 |
| Manners | W1 | FB | 0.0270 | 0.0326 | 0.9876 | 0.9764 | 0.0000 | 0.0930 |
| Manners | W2 | W3 | 0.5390 | 0.3663 | 0.9917 | 0.9607 | 0.0000 | 0.2941 |
| Manners | W2 | IFT | 0.1265 | 0.0953 | 0.9832 | 0.9432 | 0.0667 | 0.1449 |
| Manners | W2 | PB | 0.1087 | 0.0554 | 0.9858 | 0.9755 | 0.0000 | 0.0417 |
| Manners | W2 | FB | 0.0605 | 0.0400 | 0.9902 | NA | 0.0000 | NA |

| | | | | | | | | |
|---|---|---|---|---|---|---|---|---|
| Manners | W2b | IFT | 0.1470 | 0.0965 | 0.9854 | 0.9531 | 0.0000 | 0.1765 |
| Manners | W2b | PB | 0.0959 | 0.0521 | 0.9880 | 0.9652 | 0.0000 | 0.0000 |
| Manners | W2b | FB | 0.0328 | 0.0413 | 0.9864 | NA | 0.0000 | NA |
| Manners | W3 | IFT | 0.0945 | 0.0891 | 0.9797 | 0.9481 | 0.2222 | 0.1563 |
| Manners | W3 | PB | 0.0778 | 0.0475 | 0.9865 | 0.9798 | 0.0000 | 0.0536 |
| Manners | W3 | FB | 0.0313 | 0.0451 | 0.9888 | NA | 0.0000 | NA |
| Manners | IFT | PB | 0.0830 | 0.0833 | 0.9880 | 0.9734 | 0.0556 | 0.0656 |
| Manners | IFT | FB | 0.1165 | 0.1032 | 0.9896 | 0.9763 | 0.1176 | 0.0417 |
| Manners | PB | FB | 0.4992 | 0.4641 | 0.9915 | 0.9822 | 0.4444 | 0.5000 |
| Resource Guarding | P | W1 | -0.0007 | -0.0017 | NA | NA | NA | NA |
| Resource Guarding | P | W2 | -0.0027 | -0.0021 | NA | NA | NA | NA |
| Resource Guarding | P | W2b | 0.0000 | -0.0035 | NA | NA | NA | NA |
| Resource Guarding | P | W3 | 0.0000 | 0.0000 | NA | NA | NA | NA |
| Resource Guarding | P | IFT | -0.0024 | -0.0044 | NA | NA | NA | NA |
| Resource Guarding | P | PB | -0.0017 | -0.0042 | NA | NA | NA | NA |

| | | | | | | | | |
|---|---|---|---|---|---|---|---|---|
| Resource Guarding | P | FB | -0.0021 | -0.0055 | NA | NA | NA | NA |
| Resource Guarding | W1 | W2 | -0.0037 | -0.0080 | NA | NA | NA | NA |
| Resource Guarding | W1 | W2b | -0.0028 | 0.0640 | NA | NA | NA | NA |
| Resource Guarding | W1 | W3 | -0.0039 | 0.0732 | NA | NA | NA | NA |
| Resource Guarding | W1 | IFT | -0.0038 | 0.0313 | NA | NA | NA | NA |
| Resource Guarding | W1 | PB | -0.0064 | 0.0049 | NA | NA | NA | NA |
| Resource Guarding | W1 | FB | -0.0074 | 0.0110 | NA | NA | NA | NA |
| Resource Guarding | W2 | W3 | -0.0047 | 0.1502 | NA | NA | NA | NA |
| Resource Guarding | W2 | IFT | -0.0026 | -0.0026 | NA | NA | NA | NA |
| Resource Guarding | W2 | PB | -0.0051 | -0.0063 | NA | NA | NA | NA |
| Resource Guarding | W2 | FB | -0.0021 | 0.0276 | NA | NA | NA | NA |
| Resource Guarding | W2b | IFT | -0.0057 | -0.0095 | NA | NA | NA | NA |
| Resource Guarding | W2b | PB | 0.0550 | 0.0501 | NA | NA | NA | NA |
| Resource Guarding | W2b | FB | -0.0055 | -0.0094 | NA | NA | NA | NA |
| Resource Guarding | W3 | IFT | 0.2835 | 0.1469 | NA | NA | NA | NA |

| | | | | | | | | |
|---|---|---|---|---|---|---|---|---|
| Resource Guarding | W3 | PB | 0.0563 | 0.0835 | NA | NA | NA | NA |
| Resource Guarding | W3 | FB | -0.0043 | -0.0090 | NA | NA | NA | NA |
| Resource Guarding | IFT | PB | -0.0081 | -0.0069 | NA | NA | NA | NA |
| Resource Guarding | IFT | FB | -0.0079 | -0.0075 | NA | NA | NA | NA |
| Resource Guarding | PB | FB | 0.3967 | 0.5311 | NA | NA | NA | NA |
| Touch Sensitivity | P | W1 | 0.1402 | 0.0579 | 0.9991 | NA | 0.0000 | NA |
| Touch Sensitivity | P | W2 | 0.0221 | 0.0282 | 0.9983 | NA | 0.0000 | NA |
| Touch Sensitivity | P | W2b | -0.0291 | 0.0485 | 0.9922 | 0.9736 | 0.0000 | 0.0500 |
| Touch Sensitivity | P | W3 | 0.0185 | 0.0285 | 0.9949 | 0.9756 | 0.0000 | 0.0000 |
| Touch Sensitivity | P | IFT | -0.0929 | -0.0031 | 0.9905 | 0.9521 | 0.0000 | 0.0571 |
| Touch Sensitivity | P | PB | 0.1013 | 0.0191 | 0.9608 | 0.8759 | 0.2500 | 0.0968 |
| Touch Sensitivity | P | FB | 0.0700 | 0.0167 | 0.9816 | 0.9081 | 0.0000 | 0.0000 |
| Touch Sensitivity | W1 | W2 | 0.2397 | 0.1807 | 0.9967 | NA | 0.0000 | NA |
| Touch Sensitivity | W1 | W2b | 0.1492 | 0.1235 | NA | NA | NA | NA |
| Touch Sensitivity | W1 | W3 | 0.1394 | 0.0961 | 0.9965 | NA | 0.0000 | NA |

| | | | | | | | | |
|---|---|---|---|---|---|---|---|---|
| Touch Sensitivity | W1 | IFT | 0.0700 | 0.0290 | 0.9874 | NA | 0.0000 | NA |
| Touch Sensitivity | W1 | PB | 0.0246 | 0.0198 | 0.9560 | NA | 0.0000 | NA |
| Touch Sensitivity | W1 | FB | 0.0679 | 0.0115 | 0.9804 | NA | 0.0000 | NA |
| Touch Sensitivity | W2 | W3 | 0.3168 | 0.2223 | 0.9977 | NA | 0.0000 | NA |
| Touch Sensitivity | W2 | IFT | 0.1541 | 0.0996 | 0.9886 | NA | 0.0000 | NA |
| Touch Sensitivity | W2 | PB | 0.0700 | 0.0742 | 0.9605 | NA | 0.0000 | NA |
| Touch Sensitivity | W2 | FB | 0.0952 | 0.0695 | 0.9823 | NA | 0.0000 | NA |
| Touch Sensitivity | W2b | IFT | 0.0508 | 0.1261 | 0.9897 | 0.9590 | 0.2500 | 0.2381 |
| Touch Sensitivity | W2b | PB | 0.1604 | 0.0995 | 0.9616 | 0.8973 | 0.4000 | 0.3810 |
| Touch Sensitivity | W2b | FB | 0.1527 | 0.0803 | 0.9780 | 0.9154 | 0.0000 | 0.1765 |
| Touch Sensitivity | W3 | IFT | 0.0280 | 0.1052 | 0.9905 | 0.9478 | 0.5000 | 0.1905 |
| Touch Sensitivity | W3 | PB | 0.0416 | 0.0508 | 0.9601 | 0.8751 | 0.0000 | 0.4286 |
| Touch Sensitivity | W3 | FB | 0.0089 | 0.0602 | 0.9834 | NA | 0.0000 | NA |
| Touch Sensitivity | IFT | PB | 0.1111 | 0.1893 | 0.9618 | 0.9006 | 0.2222 | 0.4839 |
| Touch Sensitivity | IFT | FB | 0.1364 | 0.1623 | 0.9792 | 0.9257 | 0.1250 | 0.3571 |

| | | | | | | | | |
|---|---|---|---|---|---|---|---|---|
| Touch Sensitivity | PB | FB | 0.7243 | 0.5917 | 0.992 7 | 0.9656 | 0.5000 | 0.5621 |
